# Integration of transcriptomics and proteomics to elucidate inhibitory effect and mechanism of rosmarinic acid from *Perilla frutescens* (L.) Britt. in treating *Trichophyton mentagrophytes*

**DOI:** 10.1186/s13020-023-00772-2

**Published:** 2023-06-06

**Authors:** Yang-ding Xu, Yu-jie Guo, He-rong Mao, Zhi-xiang Xiong, Meng-yu Luo, Rui-qi Luo, Shan Lu, Lu Huang, Yi Hong

**Affiliations:** 1 School of Pharmacy, Hubei University of Chinese Medicine, Wuhan, 430065 China; 2 Guangzhou Wellhealth Bio-Pharmaceutical CO., Ltd, Guangzhou, 510200 China; 3 International Center for TCM Communication Studies, Hubei University of Chinese Medicine, Wuhan, 430065 China; 4 School of Foreign Languages, Hubei University of Chinese Medicine, Wuhan, 430065 China

**Keywords:** *Perilla frutescens*, Rosmarinic acid, *Trichophyton mentagrophytes*, Transcriptomics, Proteomics, Enolase

## Abstract

**Background:**

Dermatophyte caused by *Trichophyton mentagrophytes* is a global disease with a growing prevalence that is difficult to cure. *Perilla frutescens* (L.) Britt. is an edible and medicinal plant. Ancient books of Traditional Chinese Medicine and modern pharmacological studies have shown that it has potential anti-fungi activity. This is the first study to explore the inhibitory effects of compounds from *P. frutescens* on *Trichophyton mentagrophytes* and its mechanism of action coupled with the antifungal activity in vitro from network pharmacology, transcriptomics and proteomics.

**Methods:**

Five most potential inhibitory compounds against fungi in *P. frutescens* was screened with network pharmacology. The antifungal activity of the candidates was detected by a broth microdilution method. Through in vitro antifungal assays screening the compound with efficacy, transcriptomics and proteomics were performed to investigate the pharmacological mechanisms of the effective compound against *Trichophyton mentagrophytes*. Furthermore, the real-time polymerase chain reaction (PCR) was applied to verify the expression of genes.

**Results:**

The top five potential antifungal compounds in *P. frutescens* screened by network pharmacology are: progesterone, luteolin, apigenin, ursolic acid and rosmarinic acid. In vitro antifungal assays showed that rosmarinic acid had a favorable inhibitory effect on fungi. The transcriptomic findings exhibited that the differentially expressed genes of fungus after rosmarinic acid intervention were mainly enriched in the carbon metabolism pathway, while the proteomic findings suggested that rosmarinic acid could inhibit the average growth of *Trichophyton mentagrophytes* by interfering with the expression of enolase in the glycolysis pathway. Comparison of real-time PCR and transcriptomics results showed that the trends of gene expression in glycolytic, carbon metabolism and glutathione metabolic pathways were identical. The binding modes and interactions between rosmarinic acid and enolase were preliminary explored by molecular docking analysis.

**Conclusion:**

The key findings of the present study manifested that rosmarinic acid, a medicinal compound extracted from *P. frutescens*, had pharmacological activity in inhibiting the growth of *Trichophyton mentagrophytes* by affecting its enolase expression to reduce metabolism. Rosmarinic acid is expected to be an efficacious product for prevention and treatment of dermatophytes.

**Supplementary Information:**

The online version contains supplementary material available at 10.1186/s13020-023-00772-2.

## Introduction

In recent years, an increasing number of cases of dermatophytes have been reported, in which *Trichophyton mentagrophytes* responsible for human dermatophytosis such as tinea corpora, tinea cruris and tinea capitis [1–3]. Although many drugs like terbinafine and azoles have shown desirable curative effects in treating intractable recurrent skin tinea disease, abuse of these drugs is liable to produce drug-resistant fungi [4–6] like *Trichophyton mentagrophytes*, thus significantly reducing their therapeutic effects in clinical practice [7–9]. Another study manifested that *Trichophyton mentagrophytes* could be isolated from patients with beriberi [10]. In addition, treatment with fluconazole and other drugs contributes to an increase in 4β-hydroxycholesterol [11], which in turn negatively impacts the body.

With the development of Chinese pharmacology, an increasing number of natural bioactivity components used in combination with the commonly-used antifungal drugs can improve the susceptibility of dermatophytes to antimicrobials and even reverse drug resistance. Pharmacological studies have exhibited that natural compounds like curcumin, eugenol, magnoflorine, and geraniol can inhibit drug-resistant dermatophytes with different mechanisms from the existing antifungals [12–16]. Therefore, the use of newly-discovered natural antifungal compounds will probably mitigate the evolution of drug-resistant fungi, reduce the drug dose and improve the therapeutic efficacy in the treatment of dermatophytes. *Perilla frutescens* (L.) Britt. is an annual herb of the Labiatae family [17, 18]. The medicinal part is mainly the leaves, which have analgesic, sedative and detoxifying effects. In the Northern Song Dynasty, the *Taiping Shenghui Fang* written by Wang Huaiyin recorded the use of *P. frutescens* as the main medicine, Zisu Powder, for the treatment of women's beriberi. In addition, the *Compendium of Materia Medica* compiled by Li Shizhen, a world-renowned medical scientist in the Ming dynasty has recorded that *P. frutescens* can effectively treat diarrhea, abdominal distension, dermatophytosis, etc. In Chinese medicinals, the stems, leaves, and seeds of *P. frutescens* are commonly used as effective medicines for treating pain, colds, cough, nausea, poisoning, asthma and constipation [19]. Modern studies have shown that *P. frutescens* has anti-bacterial [20], anti-inflammatory [21–23], anti-cancer [24], anti-viral [25, 26], and anti-aging effects [27]. Most of these studies have focused on the inhibitory effects of *P. frutescens* extracts on the common bacteria and plant fungi in daily foods [20, 28]. However, studies about the effects of *P. frutescens* extracts on dermatophytes, a type of fungus present on the body surface of animals, are rather limited [29]. Investigators determined the minimum inhibitory doses and minimum fungicidal dose of *Trichophyton mentagrophytes* by using airtight boxes*,* in which *P. frutescens* extracts had a good inhibitory effect [30, 31]. Nonetheless, the potential mechanism of how the active ingredients of *P. frutescens* extracts inhibit the growth of dermatophytes remains unclear. However, the network pharmacology through the disease-target-drug interaction network, contributes to the discovery of new functions of known compounds [32].

**Fig. 1:**
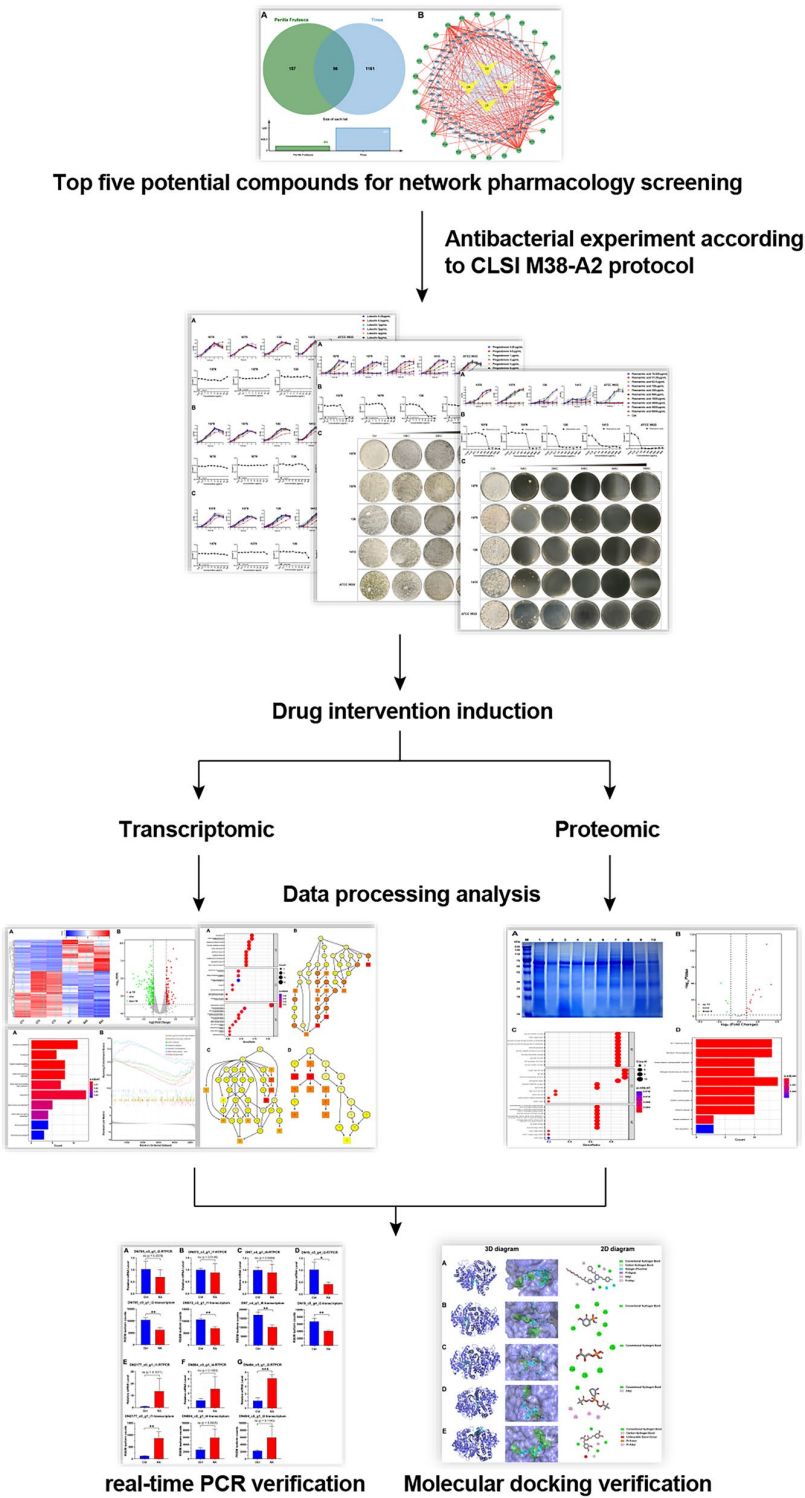
A schematic diagram of an integrated strategy of network pharmacology, transcriptomics and proteomics for revealing the mechanism of compounds extracted from *P. frutescens*

Given all the setbacks above, by utilizing network pharmacology, transcriptomics and proteomics, the present study aimed to screen the potential compounds from *P. frutescens* in inhibiting the growth of *Trichophyton* dermatophytes and elucidating the potential molecular mechanisms. In this study, firstly, network pharmacology was utilized to screen the potential compounds and then performed in vitro fungal inhibition assays to verify the therapeutic efficacy of the potential compounds. Besides, transcriptomics and proteomics were adopted to explore the potential compounds’ targets of action, and real-time PCR and molecular docking analysis were used to verify the gene expression. This study would probably provide a potentially more effective treatment for *Trichophyton mentagrophytes*. The whole study was illustrated in the flowchart of Fig. 1.

## Materials and methods

### Network pharmacology analysis

#### Active compounds collection and targets prediction

*P. frutescens* was used as a keyword to search the terms in the Traditional Chinese Medicine Systems Pharmacology Database and Analysis Platform (TCMSP) [33]. Compounds that met the following criteria were selected and considered as *P. frutescens* active ones: drug-likeness (DL) ≥ 0.18 [34]. The targets corresponding to the active compounds were extracted as *P. frutescens* targets. *P. frutescens* and subsequent tinea targets were normalized in the UniProt protein database to prevent bias caused by different nomenclatural methods [35].

#### Tinea targets collection

Tinea was used as a keyword to search in the Comparative Toxicogenomics Database (CTD) [36] to obtain tinea-related targets, and the intersection of *P. frutescens* and tinea targets was obtained by EXCEL. The Venn diagram was plotted through the online website www. bioinformatics.com.cn.

#### Drug-target-disease network construction

Cytoscape (version 3.9.0) [37] was used to construct the *P. frutescens*-target-tinea network to visualize the complex relationships between compounds, targets and diseases. Analysis Network of Cytoscape was used to perform the network characteristics analysis and to screen the major active compounds.

### Chemicals and reagents

Progesterone, ursolic acid, apigenin, luteolin and miconazole (≥ 95%) were purchased from Aladdin. Rosmarinic acid (≥ 95%) was purchased from Shanghai Puyu Kemao Co., Ltd. (Shanghai, China) and Dimethyl sulfoxide (DMSO) (≥ 99.8%) from Shandong Yousuo Chemical Technology Co., Ltd. (Linyi, Shandong, China).SDS-PAGE gel preparation kits, Prestained Protein Marker II (10–200 kDa), RPMI-1640, Radio Immunoprecipitation Assay (RIPA) lysate, Cocktail protease inhibitors, phenylmethylsulfonyl fluoride (PMSF), phosphorylation protease inhibitors, bicinchoninic acid (BCA) kit, Coomassie brilliant blue, loading buffer, Trizol, SweScript RT II First Strand cDNA Synthesis Kit and 2 × SYBR Green qPCR Master Mix were all purchased from Servicebio (Wuhan, Hubei, China). Fetal Bovine Serum (FBS) was purchased from Zhejiang Tianhang Biotechnology Co., Ltd. (Huzhou, Zhejiang, China). Potato Dextrose Agar (PDA) was purchased from Qingdao Hope Bio-Technology Co., Ltd. (Qingdao, Shandong, China) Clinical strains 1078, 1079, 130, and 1413 were shared by Wuhan No. 1 Hospital (Wuhan, Hubei, China). *Trichophyton mentagrophytes* ATCC9533 was purchased from Shanghai Bioresource Collection Center (Shanghai, China).

### Preparation of drug working solution and spore suspension

Progesterone, ursolic acid, apigenin and luteolin were dissolved using DMSO to make a final concentration of 12.8 mg/mL mother solution and then filtered for sterilization. An appropriate amount of rosmarinic acid powder was weighed and dissolved in RPMI-1640 with 10% FBS to make the final concentration of 8 mg/mL and then filtered for sterilization. *Trichophyton* dermatophytes were cultivated on PDA at 28 ºC and were periodically transferred at 60-day intervals for preservation, filtered by 8 layers of sterile gauze with an appropriate amount of distilled water, and counted by blood cell counting plates. The concentration of spore suspension was finally diluted to 2 × 10^4^ CFU/mL by adding RPMI-1640 with 10% FBS.

### Determination of minimum inhibitory concentration and minimum bactericidal concentration

The antifungal activity of the drug was tested by the broth microdilution method according to Clinical and Laboratory Standards Institute (CLSI) M38-A2. A sterile 96-well plate was taken, and 100 μL RPMI-1640 containing 10% FBS was added to the well 1 of each row as a blank control. 200 μL of rosmarinic acid working solution containing spore suspension was added to well 2. 100 μL of spore suspension was added to wells 3–11 respectively. Wells 3–11 were two-fold diluted. Well 12 in each row did not contain drugs and was used as the growth control. The drug-sensitive plates were incubated at 28 ºC for 6 days under constant temperature and light. Compared with the growth control, the minimum inhibitory concentration (MIC) was determined to be the lowest drug concentration where higher than 80% growth was inhibited. The maximum concentration of progesterone, ursolic acid, apigenin and luteolin were set as 128 μg/mL two-fold dilution, and miconazole concentration in the positive group was determined to be 8 μg/mL, and the interference was eliminated by adding DMSO of equal concentration in the control group, with DMSO concentrations ≤ 1% in all systems. The minimum bactericidal concentration (MBC) was determined by the plate recovery method, and 100 μL liquid absorbed from the integral multiples MIC wells measured on the 6th day was coated onto PDA plates and incubated at 28 ºC for 5 days. The drug concentration corresponding to the colony-free growth plate was used as MBC. All experiments were performed in three biological replicates.

### Transcriptomic analysis and data processing

After 4 h treatment with 1MIC rosmarinic acid, the mycelia of well-grown *Trichophyton mentagrophytes* were collected for transcriptomic sequencing, which was performed by Beijing Biomarker Technologies Co.,Ltd. Briefly, total RNA was extracted from the control and rosmarinic acid groups and its quality was detected, then mRNA was purified and fragmented, and finally double-stranded cDNA was synthesized using mRNA as the template. RNA sequencing library was constructed by PCR amplification. Upon completion, library quality was assessed on the Agilent Bioanalyzer 2100. After qualified library screening, TruSeq PE Cluster Kit v3-cBot-HS (Illumia) was used to cluster index-coded samples on cBot Cluster Generation System. Library preparations were then sequenced using the Illumina Hiseq 2000 and paired-end reads were generated. Trinity software was used to assemble all reads [38], and the gene expression level of each sample was estimated by RNA-Seq by Expectation–Maximization (RSEM) [39]. Specific steps are available in Additional file 1: Method 1. Sequencing results have been uploaded to the National Center for Biotechnology Information, and sequence number PRJNA847061 provides access to all RNA-seq data.

In this experiment, the DESeq R package was used to analyze differentially expressed genes (DEGs) in the two samples. In the process of DEGs analysis, the recognized and effective Benjamini–Hochberg method was used to correct the P-value of significance obtained from the original hypothesis test, and the corrected P-value was finally adopted. False Discovery Rate (FDR) was used as a key indicator for DEGs screening to reduce the false positives caused by independent statistical hypothesis testing of the expression values of numerous genes. In the screening process, FDR less than 0.05 and Fold Change (FC) greater than or equal to 2 were considered as the screening criteria. Here, FC represents the ratio of expression between two samples or groups. Functional annotation of DEGs was performed using eggNOG [40], Gene Ontology (GO, http://www.geneontology.org/) and Kyoko Encyclopedia of Genes and Genomes (KEGG, http://www.genome.jp/kegg/) databases, enrichment analysis used the clusterProfiler R package [41], the heat map and volcano plot used gplots and ggplot2 R package respectively.

### Proteomic analysis and data processing

Rosmarinic acid working solution was added to the 5-day cultured *Trichophyton mentagrophytes* solution to adjust the concentration to 1 MIC, and RPMI-1640 containing 10% FBS was used as the control. The samples were incubated at 28 ºC for 4 h with constant temperature shock, then centrifuged at 5000 × g for 15 min. After the supernatant was discarded, the samples were washed with ultrapure water 3 times. RIPA lysate containing, coupled with its instructions specified Cocktail protease inhibitors, PMSF and phosphorylation protease inhibitors were then added, and the samples were centrifuged at 12,000 × *g* for 15 min after shaking for 10 min on ice. After BCA kit was used for protein quantification, the lowest protein concentration sample was used as the reference to adjust the protein concentration of each sample, and bovine serum protein was used as the standard. After adding loading buffer, the samples were boiled in water bath for 5 min, followed by SDS-PAGE of 8% separation glue and 5% concentration glue. Finally, Coomassie brilliant blue was used for staining after electrophoresis.

Strip sequencing was performed by Biotree Biotech Co.,Ltd. (Shanghai, China). In brief, the bands on SDS-PAGE were cut off, decolorized and dehydrated, and the polypeptide chains were extracted after trypsin digestion, dried and desalted by C18 column, and then analyzed by nanoLC-MS/MS. The off-machine data were searched and analyzed by Proteome Discoverer (version 2.4.0.305, Thermo Fisher Scientific) and the built-in Sequest HT search engine. After the abundance was normalized, differential expression was calculated, and clusterProfiler and ggplot2 R package were used for enrichment analysis and mapping. Details are available in Additional file 1: Method 2.

### Quantitative real-time PCR assays

The well-grown *Trichophyton mentagrophytes* were treated with 1 MIC rosmarinic acid for 4 h to collect mycelia. After grinding with liquid nitrogen, the RNA samples were extracted using Trizol. SweScript RT II First Strand cDNA Synthesis Kit was used to reverse transcribe RNA to obtain cDNA. Amplification was performed using 2 × SYBR Green qPCR Master Mix on qTower 3G Real-time PCR Instrument (Analytik Jena, Germany). PCR reaction conditions were as follows: 40 cycles of predenaturation at 95 ºC for 30 s, denaturation at 95 ºC for 15 s, annealing at 65 ºC for 30 s, and extension at 72 ºC for 30 s. 18S RNA gene was used as reference, and the differential expression levels of target genes and reference genes were calculated by 2^(−ΔΔCt)^ method. All primer sequences used in this experiment are available in Additional file 1: Table S1.

### Molecular Docking Studies

**Fig. 2:**
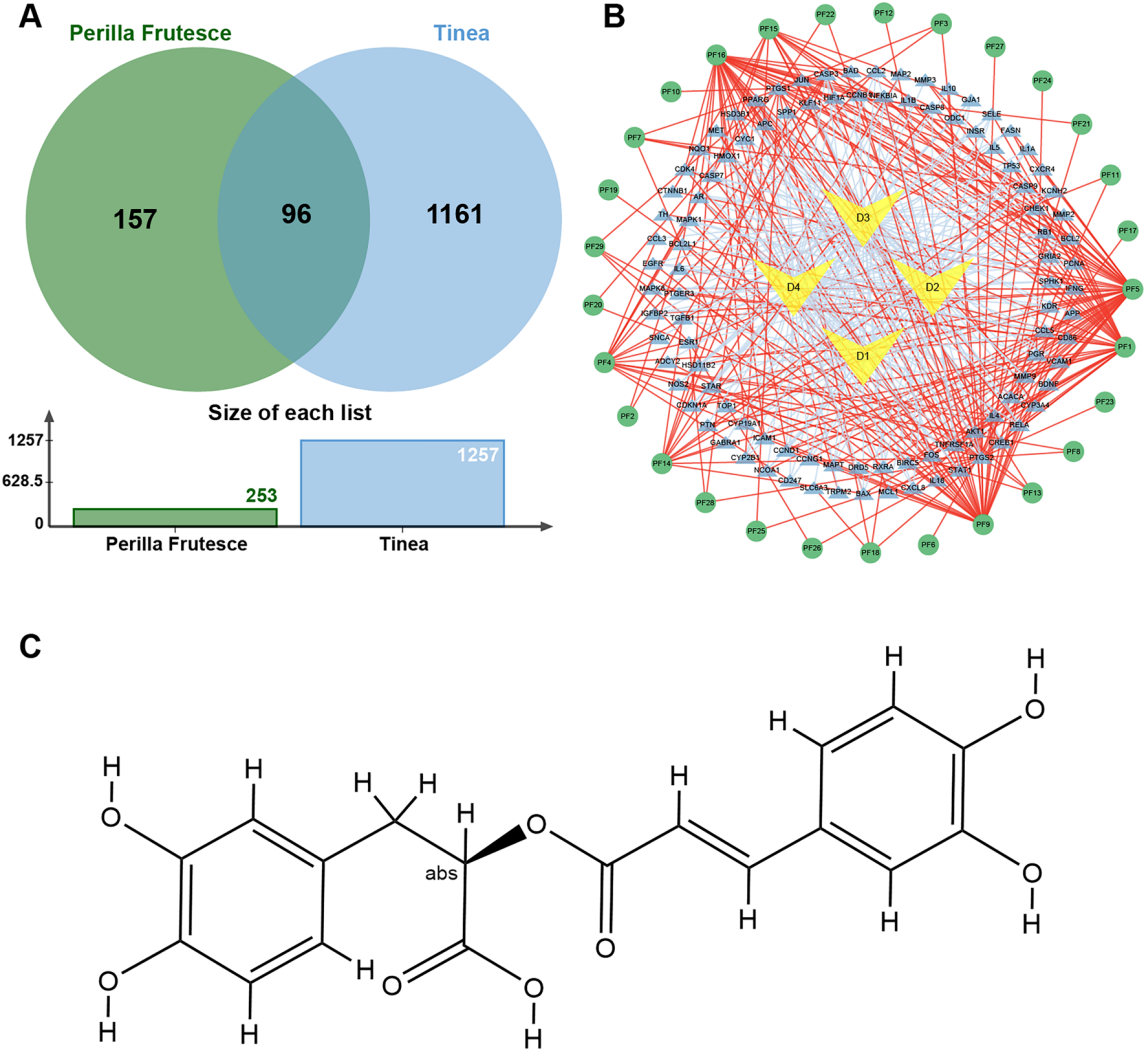
Venn diagram and *P. frutescens*-Target-Tinea network. **A** Green circles indicate all targets of *P. frutescens* action; blue circles indicate all targets of tinea disease action; the intersection of the two circles is the overlapping targets. **B** Green circles represent compounds from *P. frutescens*; blue triangles indicate disease targets; yellow quadrilaterals indicate tinea disease. **C** Molecular structural formula of rosmarinic acid (PubChem CID: 5281792)

Virtual molecular docking was employed to analyze the potential binding modes of enolase and rosmarinic acid (Fig. 2C). Four recognized enolase inhibitors were used for comparison [42–45]. All compound structures were obtained from the PubChem database (https://pubchem.ncbi.nlm.nih.gov/). AP-III-a4 is nonsubstrate analog that directly binds to enolase and inhibits its activity. Hex and D-(-)-3-Phosphoglyceric acid disodium are substrate-competitive enolase inhibitors and POMHEX increases the permeability of HEX into cells and tissue. The crystal structure of enolase was downloaded from the Uniport, which was selected and saved as pdb format. The ligand and receptor were split by Pymol. Autodock Tools 1.5.7 was used to transform pdb to pdbqt format files with gird boxes adjusted to cover the entire pocket for the preparation of virtual docking. The structures of chemicals were collected from Pubchem as sdf format and transformed into pdbqt format [46]. Autodock vina 1.2.0 was used to simulate the potential interactions among the selected compounds and the targets [47].

### Statistical analysis

The data were analyzed using GraphPad Prism statistical software. All results are presented as mean ± SEM deviation. One-way analysis of variance was performed between multiple groups when the homogeneity of variance and normality were met. P < 0.05 was considered statistically significant.

## Results

### Screening of *P. frutescens* active ingredients and targets and establishment of the drug-target-disease network

328 *P. frutescens* compounds were screened from TCMSP. Twenty-nine active compounds were screened by DL parameters (Additional file 1: Table S2). The targets corresponding to the active compounds were extracted and entered into EXCEL, with removed duplicate entries, 253 *P. frutescens* targets were obtained after merging.

A total of 1,262 Tinea targets were collected through the CTD database, and several different Tinea diseases were obtained by Tiena search (Additional file 1: Table S3). A total of 99 related targets were obtained through the mapping of *P. frutescens* and Tinea targets. Intersection of *P. frutescens* and Tinea targets was then plotted into the Venn diagram (Fig. 2A).

In the *P. frutescens*-target-Tinea network (Fig. 2B), the Degree of a single target is the number of the connected nodes. The network analysis was made by the Cytoscape, and the active compounds were sorted by Degree. The top 5 active compounds, namely, progesterone, luteolin, apigenin, ursolic acid and rosmarinic acid were listed by Betweenness Centrality, Closeness Centrality and Degree (Additional file 1: Table S4).

### Antifungi activity analysis

**Fig. 3:**
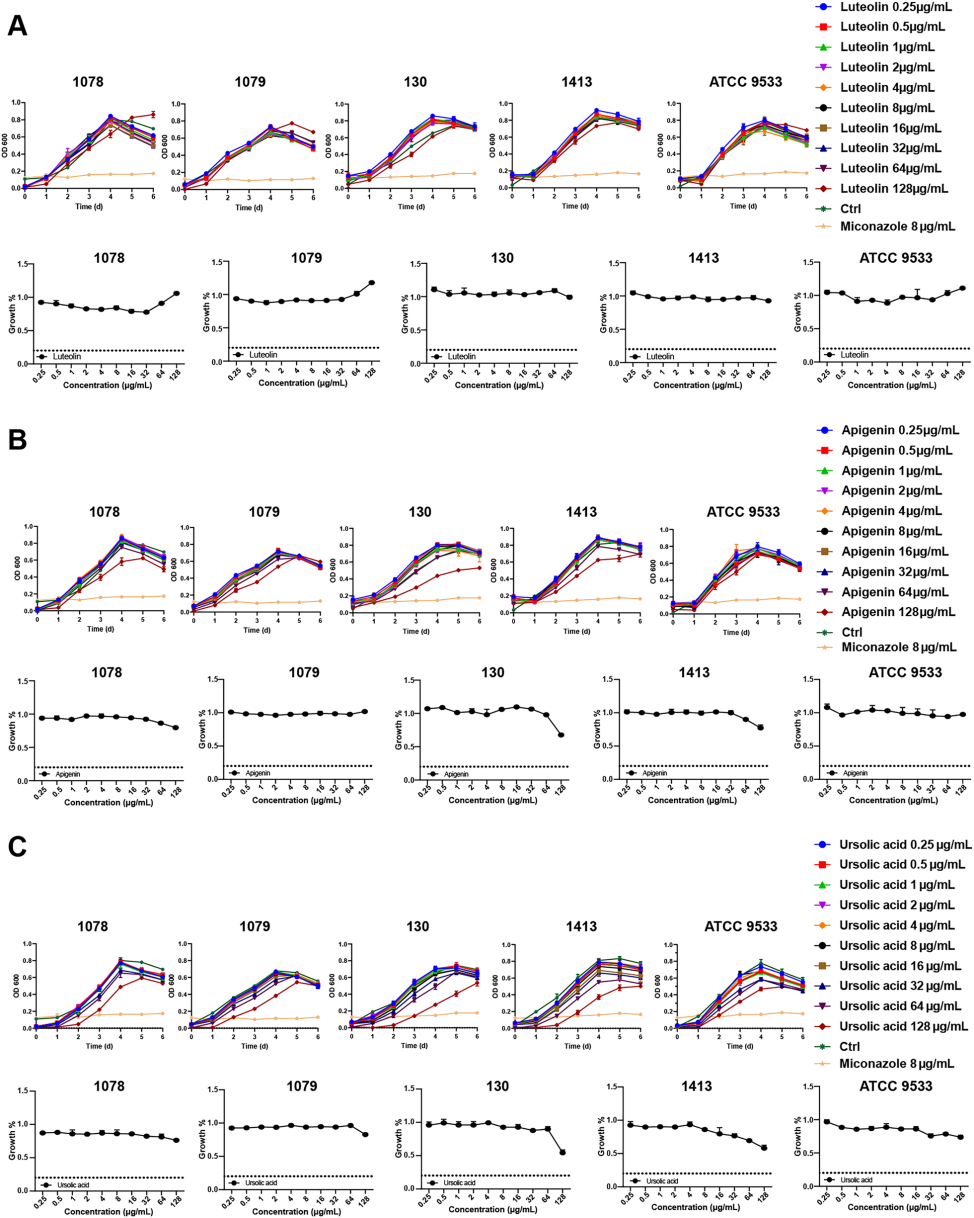
Inhibitory effects of luteolin, apigenin, and ursolic acid on *Trichophyton* dermatophytes. **A** Growth curve and inhibition rate of dermatophytes under the action of luteolin. **B** Growth curve and inhibition rate of *Trichophyton* dermatophytes under the act of apigenin. **C** Growth curve and inhibition rate of *Trichophyton* dermatophytes under the action of ursolic acid. Data were shown as mean ± SEM (n = 3)

**Fig. 4:**
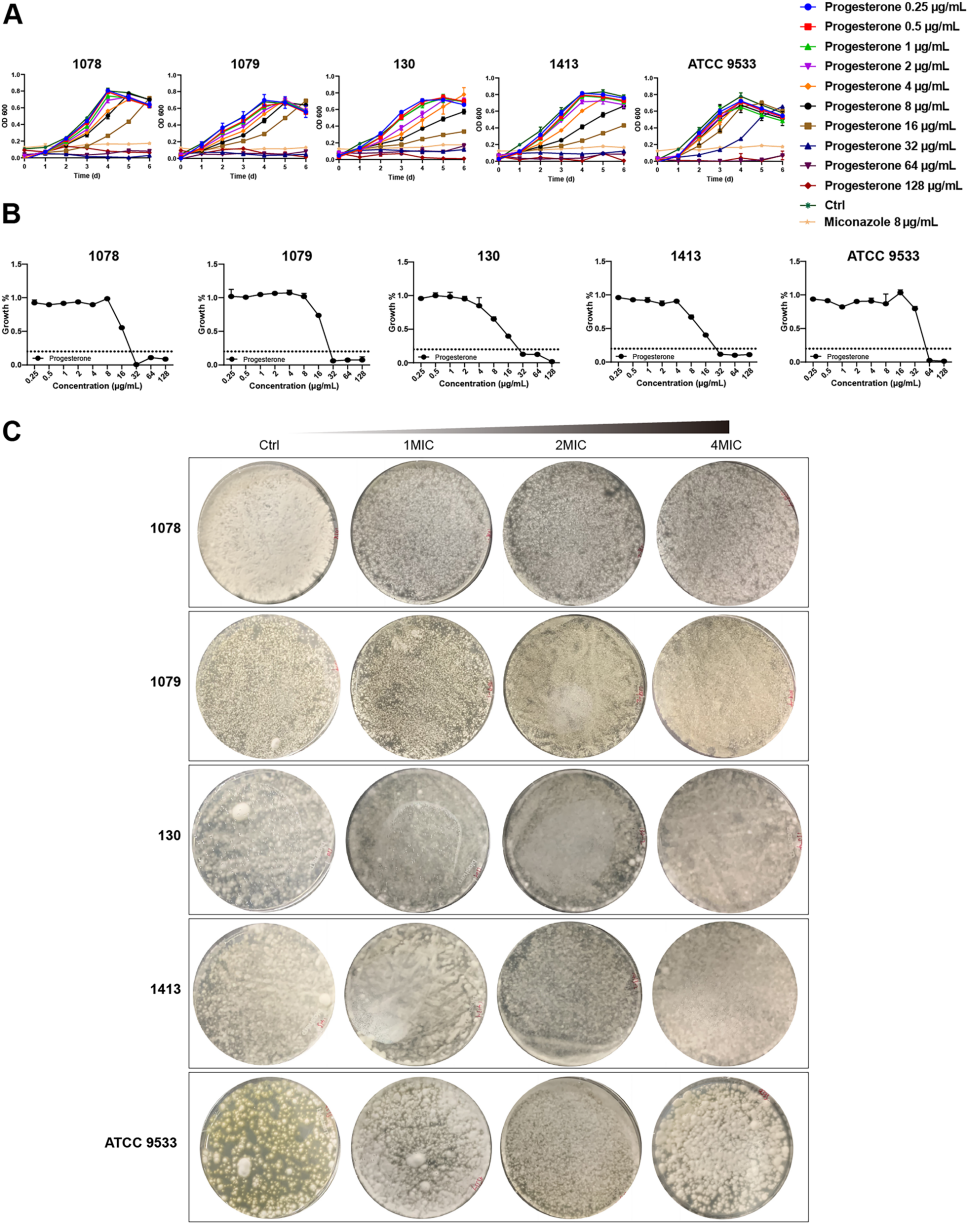
Inhibitory effect of progesterone on *Trichophyton* dermatophytes. **A** Growth curve, with an increase of progesterone concentration, the fungal growth was gradually inhibited. **B** Inhibition rate, the ratio of each concentration of progesterone treatment group to control group on the sixth day. **C** Fungal growth on the plate after six days by each concentration of progesterone treatment, there is no significant difference in the fungicidal effect after the increase of progesterone concentration. Data were shown as mean ± SEM (n = 3)

**Fig. 5:**
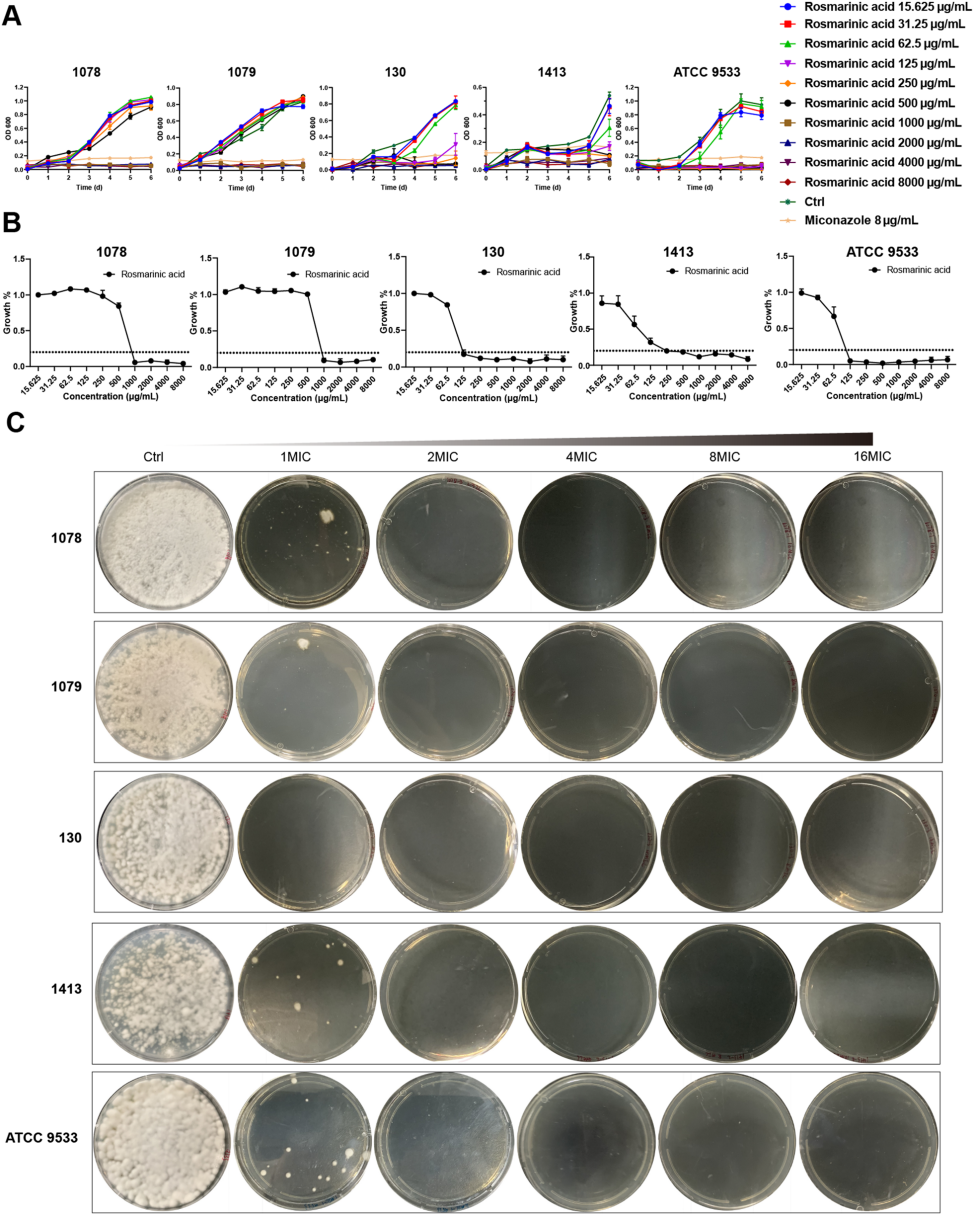
Inhibitory effect of rosmarinic acid on *Trichophyton* dermatophytes. **A** Growth curve, with increasing concentration of rosmarinic acid, the growth of the fungi was gradually inhibited. **B** Inhibition rate, the ratio of each concentration of rosmarinic acid treatment group to the control group on the sixth day. **C** Fungal activity after six days of growth on the plates treated with each concentration of the drug, with increasing concentrations of rosmarinic acid, its fungicidal activity gradually increased. Data were shown as mean ± SEM (n = 3)

In this experiment, MIC refers to the drug concentration at which the growth rate after drug treatment was less than 20% of the blank group. The inhibitory effect of luteolin on *Trichophyton* dermatophytes was shown in Fig. 3A, which revealed no significant inhibitory effect on all five experimental fungi. Ursolic acid and apigenin demonstrated similar inhibitory effects on *Trichophyton* dermatophytes (Fig. 3B, C). With increasing concentrations, the growth rate of 130 and 1413 slowed down but had no significant inhibitory effect. The inhibitory effect of progesterone on the experimental fungi was shown in Fig. 4, with MIC values of 32 μg/mL for 1078, 1079, 130 and 1413 and 64 μg/mL for ATCC 9533 in a dose-dependent manner (Fig. 4A, B). It was considered that the drug concentration for no colony growthte incubation, it was found that colonies still grew on the plate, indicating that progesterone could only inhibit fungal growth, but had no bactericidal activity. The inhibitory effect of rosmarinic acid on the experimental fungi was shown in Fig. 5 in a dose-dependent manner, with MIC values of 1000 μg/mL for 1078 and 1079, 125 μg/mL for 130 μg/mL and 250 μg/mL for 1413 (Fig. 5A, B). MBC values of 2000 μg/mL for rosmarinic acid against 1078 and 1079, 250 μg/mL for 130 and 250 μg/mL for 1413 were observed after plate incubation (Fig. 5C).

### Transcriptomic analysis

To explore the inhibitory mechanism of *Trichophyton mentagrophytes* by rosmarinic acid, RNA-seq was used to identify differential mRNA expression. The base quality values measured by Illumina sequencing platform were expressed as Phred scores (Additional file 1: Fig. S1 and Table S5). The quality score of reads was Q30 (base recognition accuracy ≥ 99.9%) and the error rate of single base sequencing was < 0.1% (Additional file 1: Table S6). Since the first few bases at the 5' end of the reads were random primer sequences with some bias, there was a significant fluctuation in the front end of the base distribution map (Additional file 1: Fig. S2)0.15,946 Unigene and 38,006 transcripts were obtained using Trinity assembly (Additional file 1: Fig. S3 and Table S7). A total of 32,798 annotated transcripts were obtained by comparing transcripts with eggNOG database using the eggNOG-Mapper tool (Additional file 1: Table S8). The reads obtained by sequencing were compared with Unigene library with Bowtie. Based on the comparison results, RSEM was used to estimate the expression level, and the expression abundance of the corresponding Unigene was represented by the trimmed mean of M values value (Additional file 1: Fig. S4, S5).

**Fig. 6:**
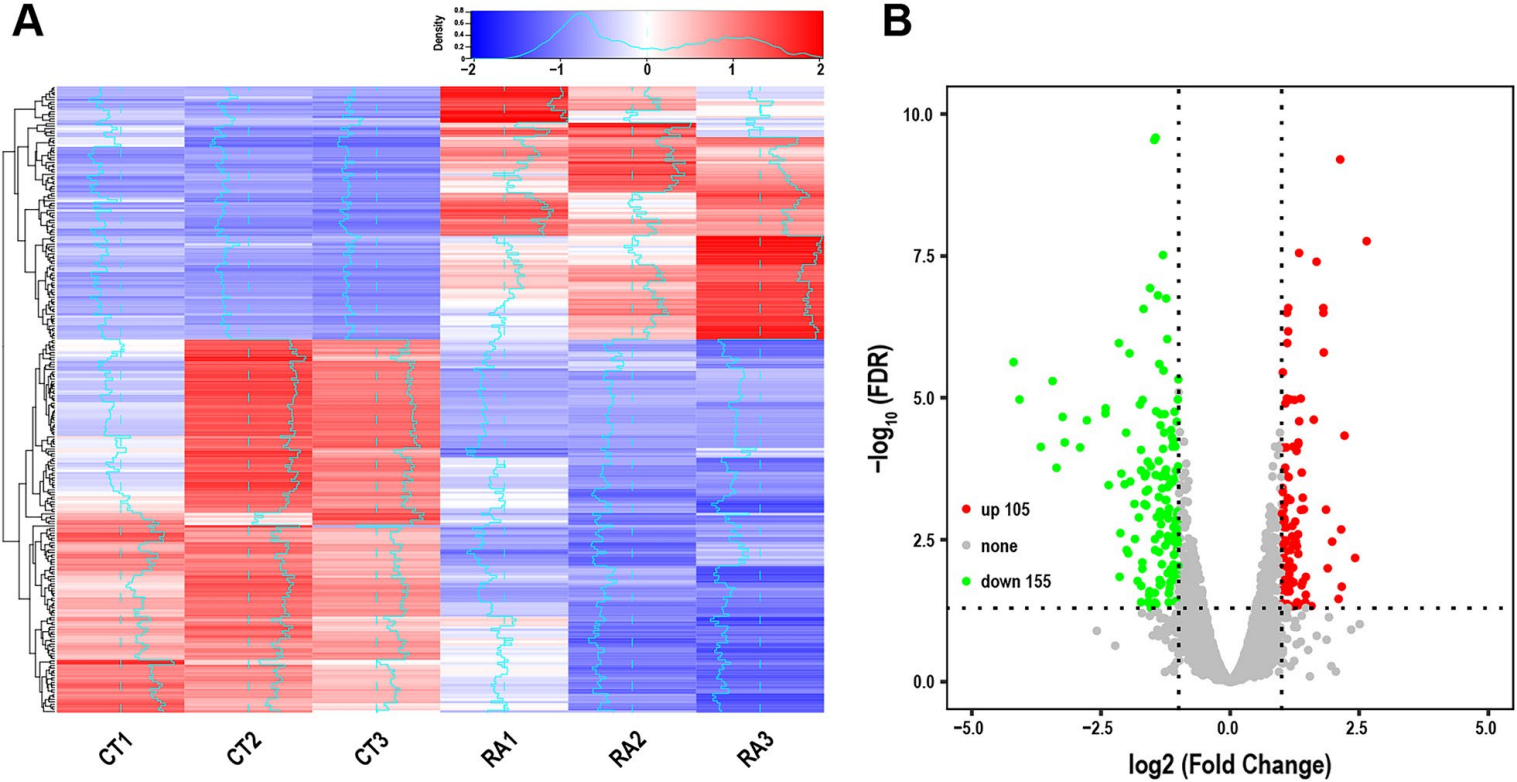
Screening of differentially expressed genes. **A** Cluster heat map of differentially expressed genes; CT1, CT2 and CT3 denote the three groups of normally growing ATCC 9533; RA1, RA2 and RA3 denote the three groups of ATCC 9533 using rosmarinic acid intervention. **B** The volcano plot was obtained by DESeq2 computational differential expression analysis of the control and rosmarinic acid treatment group with 105 genes up-regulated in expression (red) and 155 genes down-regulated in expression (green)

There were significant differences in gene expression between the control group and the rosmarinic acid treatment group. The genes were clustered according to the same or similar expression patterns (Fig. 6A), where the color-key abscissa bar indicates the multiple difference of the normalized genes and the larger absolute value indicates the more obvious differential expression. The ordinate represents the density of differential gene enrichment in the corresponding value and the trend line in the heat map represents the degree of gene deviation from the median value. The volcano plot showed the differential gene expression versus priority, where each point represents a gene, and the negative value of the logarithm of the difference Fold (horizontal coordinate: log_2_ Fold Change) indicates a decrease of gene expression, while the positive number indicates an increase (Fig. 6B and Additional file 1: Table S9). The larger logarithm of error incidence (vertical coordinate: log_10_ FDR) indicates higher confidence in the results.

**Fig. 7:**
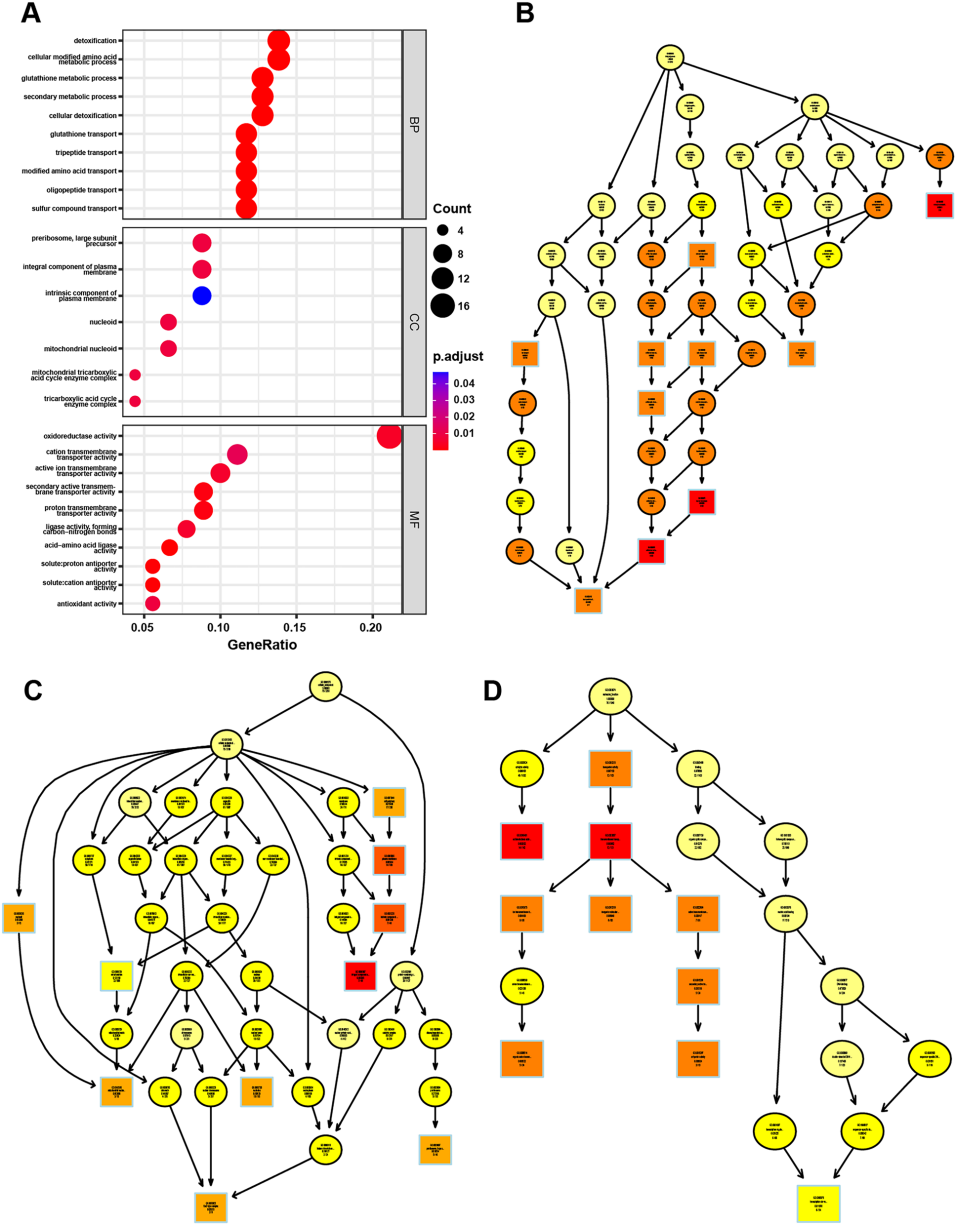
GO enrichment. **A** Biological process, cellular component and molecular function enrichment results. **B** BP-directed acyclic graph. **C** CC-directed acyclic graph. **D** MF-directed acyclic graph

DEGs were mapped to the GO database. The enrichment results of DEGs were obtained from Biological Process (BP), Cellular Component (CC), and Molecular Function (MF) of GO terms (Fig. 7A). There were 110 statistically significant GO terms, including 83 BP terms, 7 CC terms and 20 MF terms. DEGs in BP were mainly enriched in detoxification, cell modified amino acid metabolism and glutathione metabolism; DEGs in CC were mainly enriched in plasma membrane, mitochondrial nuclear mass and integral components of mitochondrial tricarboxylic acid cycle enzyme complex; DEGs in MF were mainly enriched in oxidoreductase activity, cation transmembrane transporter activity and proton transmembrane transporter activity.

The topGO R package was used to rank the pathways to visualize the GO nodes and their hierarchical relationships for differential gene enrichment. The most significant nodes were the hexose catabolic process, siderophore metabolic process, and iron assimilation under the homeostatic process. Based on the directed acyclic graph of CC, the most significant DEGs enrichment nodes were found in mitochondrial nucleocapsid and plasma membrane components. The directed acyclic graph of MF showed that the main enriched nodes of DEGs were oxidoreductase activity and transmembrane transporter activity (Fig. 7B–D).

**Fig. 8:**
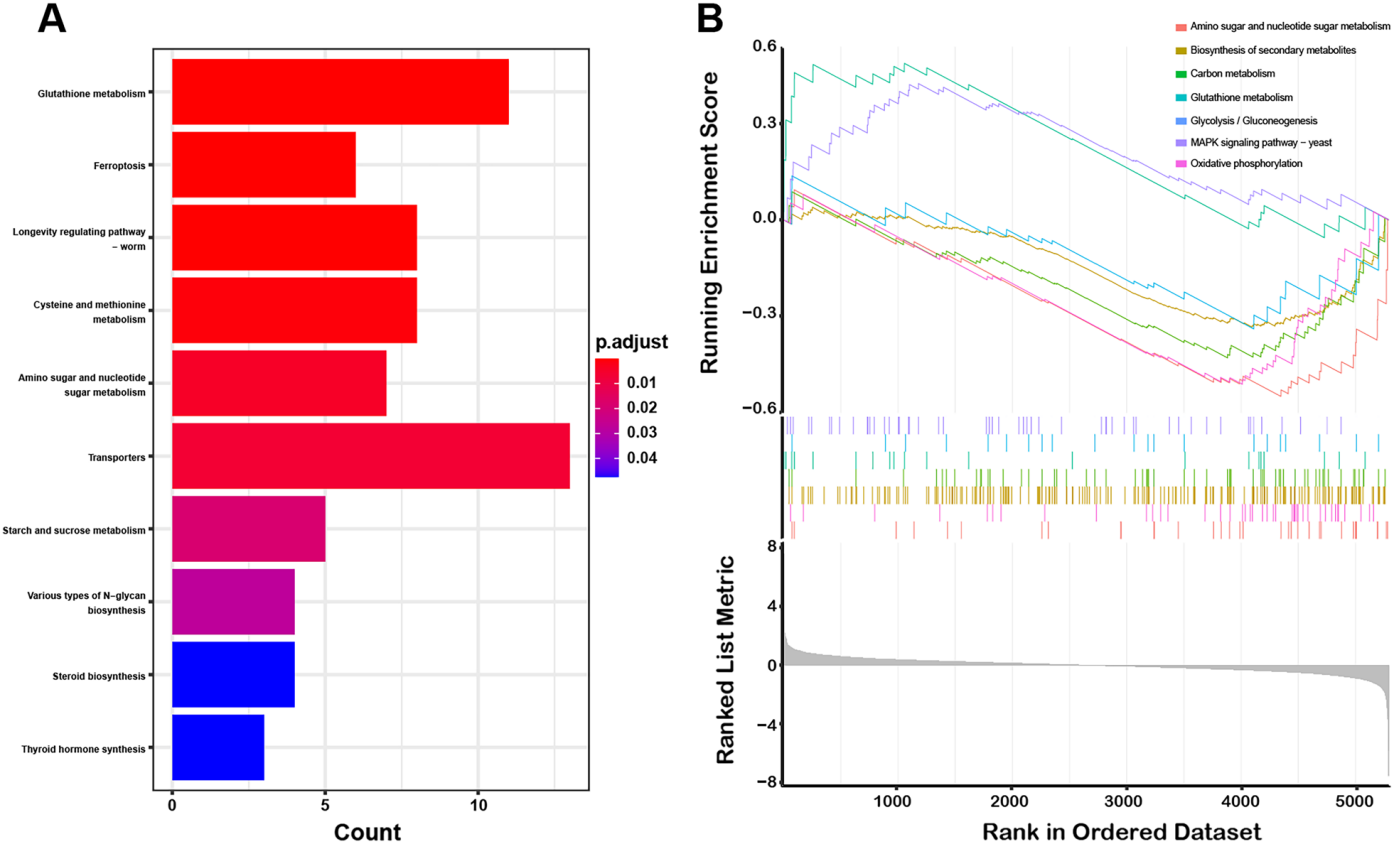
KEGG and GESA enrichment. **A** Top 10 KEGG enrichment pathways. **B** GESA analysis of critical pathways

Figure 8A showed the top 10 pathways of KEGG pathway terms, most of which were related to metabolisms, such as the metabolisms of Glutathione, cysteine, methionine, amino sugar, nucleotide sugar, starch and sucrose. The significantly enriched metabolic pathways in KEGG were selected and analyzed by Gene Set Enrichment Analysis. The results exhibited that the expression of genes enriched in glutathione metabolic pathway in the blank group was higher than that in the rosmarinic acid treatment group, while the expression levels of genes enriched in other metabolic pathways, such as amino acid sugar and nucleotide sugar metabolism, biosynthesis of secondary metabolites and carbohydrate metabolism, were lower in the blank group than in the rosmarinic acid treatment group (Fig. 8B).

### Proteomic analysis

**Fig. 9:**
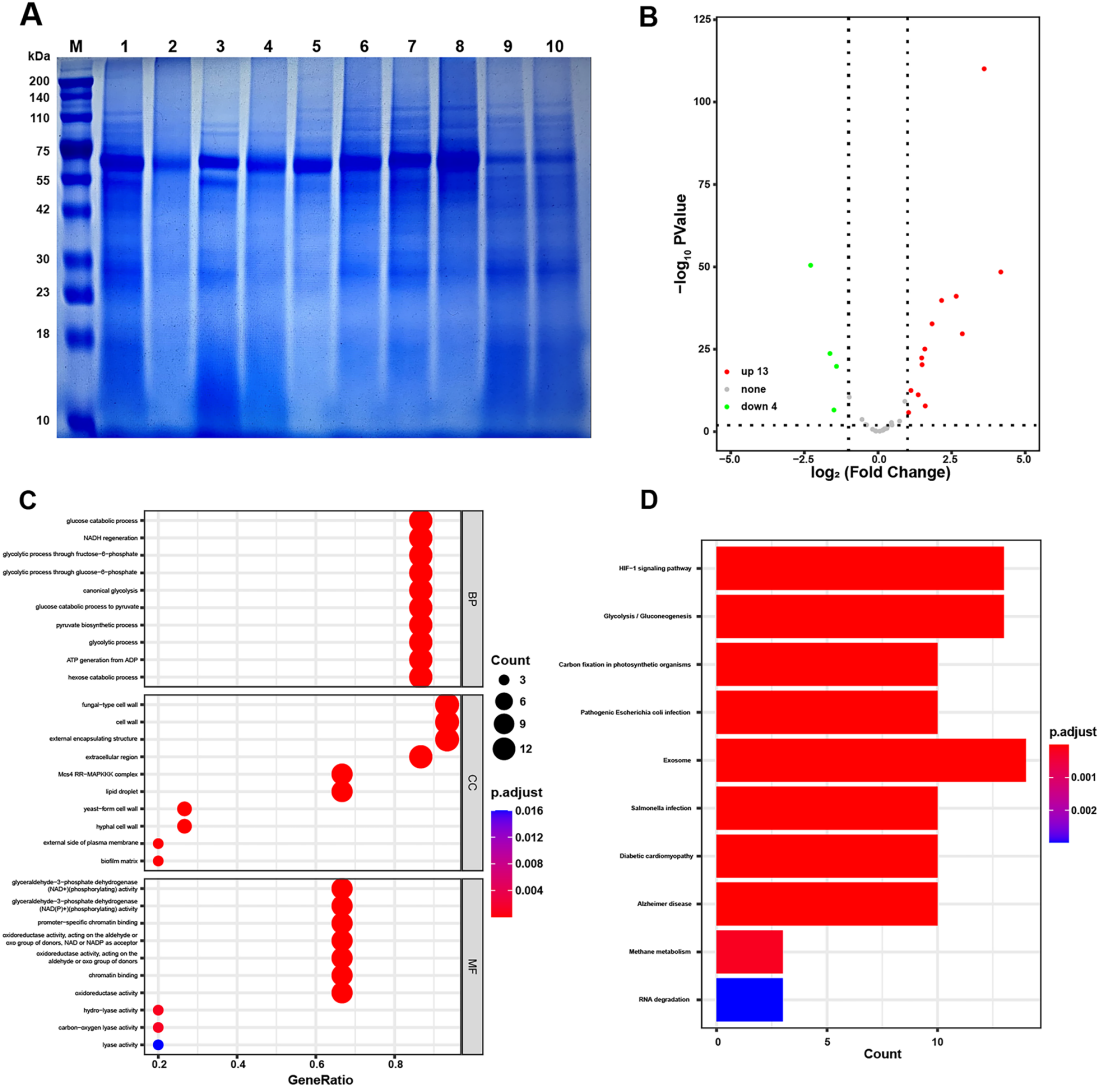
Proteomic results. **A** Total fungal protein SDS-PAGE. Lanes 1, 3, 5, 7, 9 are 1078, 1079, 130, 1413, and ATCC 9533 in the control group; lanes 2, 4, 6, 8, 10 are 1078, 1079, 130, 1413, and ATCC 9533 treated with 1MIC rosmarinic acid in the treatment group. **B** Volcano plot. Expression of 13 proteins is up-regulated (red), and Expression of 4 proteins is down-regulated (green). **C** BP, CC and MF in GO enrichment. **D** KEGG pathway enrichment

A large number of protein bands were found to disappear or appear around 75 kDa, 55 kDa, 42 kDa, and 30 kDa after treatment with rosmarinic acid compared to the control group by SDS-PAGE electrophoresis (Fig. 9A), which was possibly related to the inhibition of fungal growth caused by the increased or decreased expression of these proteins. SDS-PAGE subjected to qualitative proteomic assays further clarifies the mechanism of rosmarinic acid action. 17 of all 35 proteins were identified as differentially expressed, including 4 down-regulated proteins and 13 up-regulated ones (Fig. 9B).

The GO and KEGG pathway analysis were then used to further investigate the mechanism of rosmarinic acid against *Trichophyton mentagrophyte*s (fold change ≥ 2 and P < 0.05). There were 221 statistically significant GO terms in Fig. 9C, including 190 BP terms, 20 CC terms and 11 MF terms. Differential proteins in BP were mainly enriched in glucose catabolism, NADH regeneration, fructose-6-phosphate glycolysis and glucose-6-phosphate glycolysis; differential proteins in CC were mainly enriched in fungal cell wall, cell wall, outer envelope structure and extracellular region; the differential proteins in MF were mainly enriched in glyceraldehyde-3-phosphate dehydrogenase (NAD +) (phosphorylation) activity, glyceraldehyde-3-phosphate dehydrogenase (NAD (P) +) (phosphorylation) activity and promoter specific chromatin binding and oxidoreductase activity.

Among the top 10 pathways of KEGG pathway terms (Fig. 9D), the glycolysis/glucose production pathway was highlighted. The significantly down-regulated differentially expressed protein X5CH36 in Additional file 1: Table S10 was shown on Uniport as triglyceride 2-phosphodehydrase, another name for enolase.

### Real-time PCR verification results

**Fig. 10:**
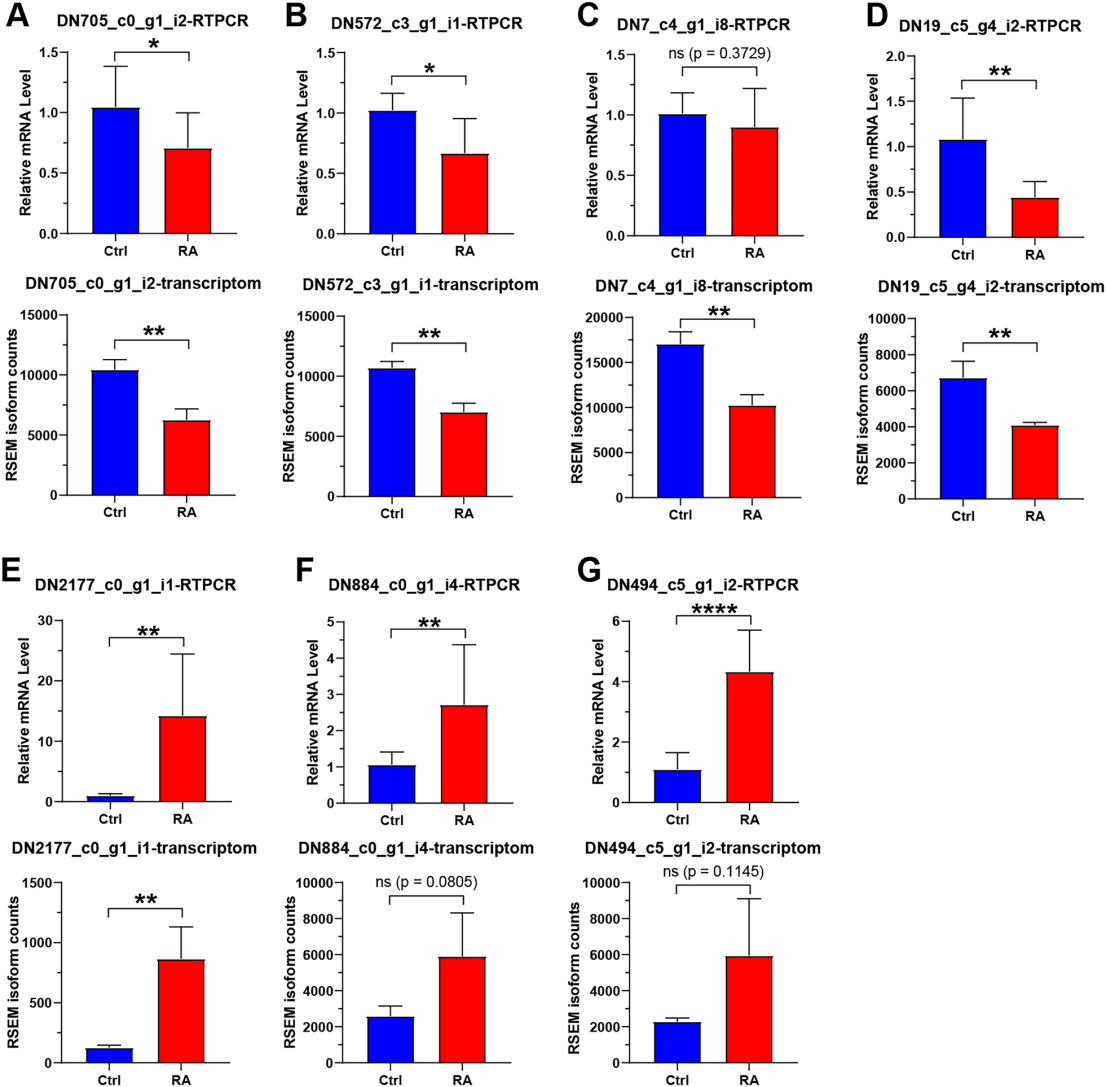
Real-time PCR. **A** The DN705_c0_g1_i2 gene. **B** The DN572_c3_g1_i1 gene. **C** The DN7_c4_g1_i8 gene. **D** The DN19_c5_g4_i2 gene. **E** The DN2177_c0_g1_i1 gene. **F** The DN884_c0_g1_i4 gene. **G** The DN494_c5_g1_i2 gene. Significant differences were found between the treatment group and the control group. Data were shown as mean ± SEM (n = 3). ^*^P < 0.05, ^**^P < 0.01, ^****^P < 0.0001

Comparison of real-time PCR and transcriptomics results showed that the expression trends of genes in glycolytic, carbon metabolism and glutathione metabolic pathways were identical (Fig. 10). Real-time PCR results of genes in the rosmarinic acid treatment group showed significant low or high fold changes compared with the control group, although no significant differences between these groups were observed in some transcriptomics results (Fig. 10). Therefore, the transcriptomics results can be considered reliable.

### Molecular docking studies

**Fig. 11:**
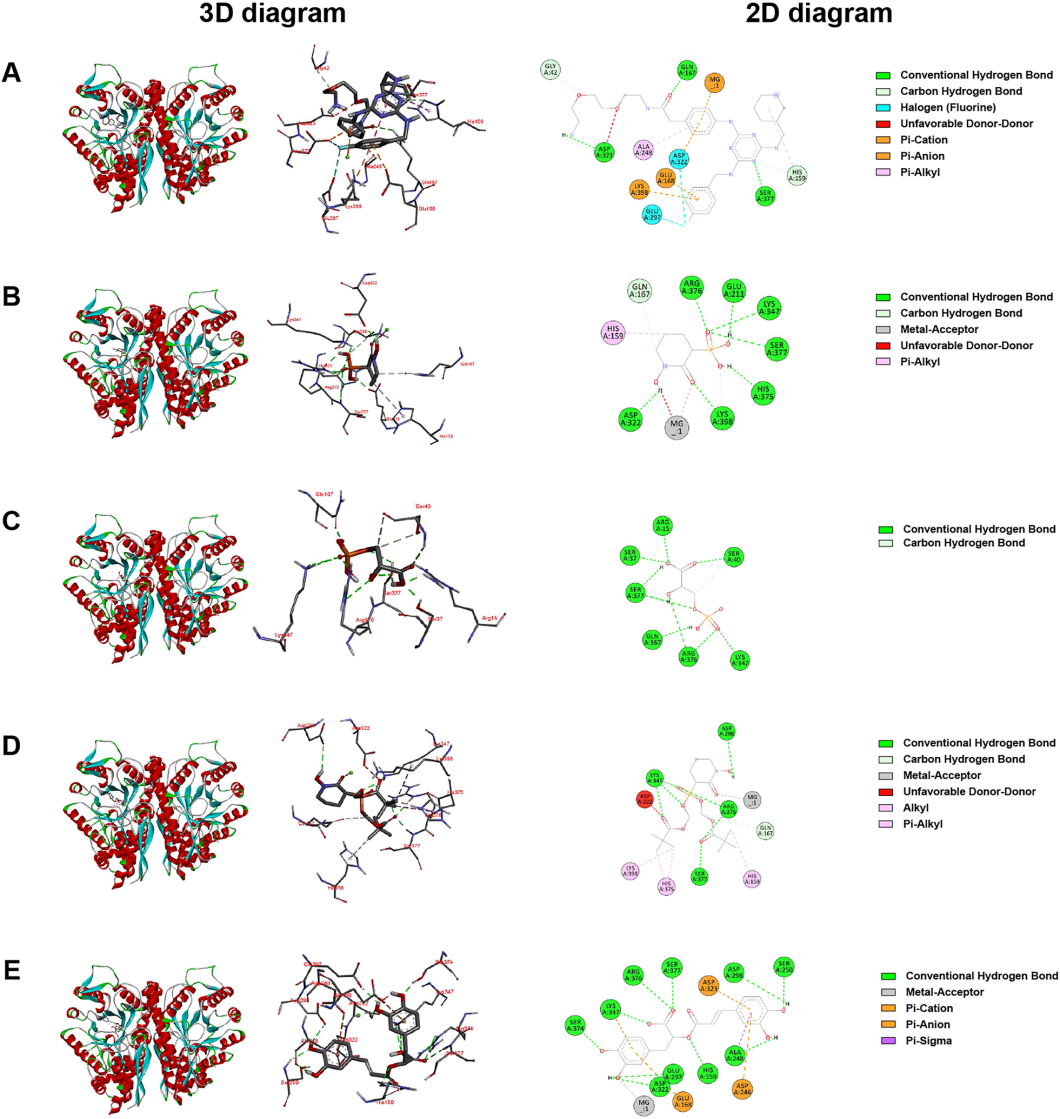
Binding modes and interactions. **A** AP-III-a4. **B** Hex. **C** D-(-)-3-Phosphoglyceric acid disodium. **D** POMHEX. **E** Rosmarinic acid

Molecular docking was used to explore the possible binding sites and modes of action between enolase and the active compounds including AP-III-a4, Hex, D-(-)-3-Phosphoglyceric acid disodium, POMHEX and rosmarinic acid. As shown in Table 1, the binding energies of Hex, POMHEX and Rosmarinic acid with enolase were all less than − 5.0 kcal/mol, indicating their good binding affinity with enolase. Moreover, rosmarinic acid showed better binding affinity than AP-III-a4, Hex, D-(-)-3-Phosphoglyceric acid disodium and POMHEX. The 3D and 2D interaction diagrams of the compounds with enolase were shown in Fig. 11.

**Table 1:** Molecular docking results of enolase and the compounds

Compounds	Binding Energy (kcal/mol)	Hydrogen Bonds	Other Amino Acid Residues	Metal ion
AP-III-a4	23.01	GLY42, HIS159, GLN167, GLU297, ASP322, ASP323,SER377	GLU168, ALA248, LYS398, Mg^2+^	–
Hex	− 5.88	GLN167, GLU211, ASP322, LYS347, HIS375, ARG376,SER377,LYS398	HIS159-	Mg^2+^
D-(-)-3-Phosphoglyceric acid disodium	− 3.2	ARG15, SER37, SER40, GLN167, LYS347, ARG376,SER377	–	–
POMHEX	− 5.88	GLN167, ASP298, LYS347, ARG376, SER377	HIS159, HIS375, ASP322, LYS398	Mg^2+^
Rosmarinic acid	− 6.75	HIS159, ALA248, SER250, GLU297, ASP298, ASP322, LYS347, SER374, ARG376, SER377	GLU168, ASP246, ASP323	Mg^2+^

## Discussion

Although many drugs are clinically available to treat dermatophytes, the emergence of drug-resistant fungi has reduced efficacy of existing drugs and even caused recurrent skin tinea disease. This may be caused by the massive abuse of existing drugs, or related to the fact that mutations in genes in the ergosterol pathway cause their resistance [8]. Therefore, it is necessary to discover more effective compounds from Traditional Chinese medicine to provide new treatment protocols. However, the complex composition of Traditional Chinese medicine makes it challenging to discover such effective compounds. With the rapid development of genomic technologies such as genomics, transcriptomics, proteomics and metabolomics, it has become possible to elucidate complex biological phenomena and obtain large amounts of genetic data, which can help explore natural compounds from Traditional Chinese medicine in treating dermatophytes [48]. In this study, the TCMSP database was used to screen potentially active compounds and transcriptomics and proteomics to make predictions about the inhibitory mechanisms of *Trichophyton mentagrophytes* growth.

*P. frutescens* is a medicinal and edible plant widely distributed in subtropical regions. *P. frutescens* is used as a medicine in China, Japan and Southeast Asian countries due to its significant medicinal functions and minor adverse effects. Rosmarinic acid is the most abundant bioactive polyphenolic component of *P. frutescens* leaves [49]. The important effects of rosmarinic acid and its derivatives on human health have been demonstrated in quite several in vitro/in vivo pharmaceutical and clinical studies. In recent years, the biological activity of rosmarinic acid has drawn widespread attention from the academic community, as it doesn’t merely inhibit the growth of methicillin-resistant *Staphylococcus aureus*, but also the growth of other drug-resistant fungi through its combined use with antibiotics. Besides, studies have also exhibited that rosmarinic acid has certain inhibitory effects on some fungi [28]. This study found that rosmarinic acid from *P. frutescens* could inhibit the average growth of *Trichophyton mentagrophytes* by affecting their metabolism.

Inhibition assays in vitro are commonly used to assess the ability of drugs of inhibiting microorganisms. In line with other studies, MIC and MBC are the main parameters for evaluating the antimicrobial ability of drugs [50]. In this study, among five compounds screened by network pharmacology: progesterone, luteolin, apigenin, ursolic acid and rosmarinic acid, only progesterone and rosmarinic acid exhibited desired antifungal ability in vitro. The results of MIC and spot test indicated that progesterone has a lower MIC but no MBC (Fig. 4), which is consistent with the previous study[51]. However, the rosmarinic acid could not only inhibit the growth of *Trichophyton mentagrophytes*, which is one of filamentous fungi, in a dose-dependent manner, but also killed it at a concentration of 2MIC (Fig. 5). The previous studies indicated the rosmarinic acid could play a significant role in inhibiting the growth of filamentous fungi, and filamentous fungi treated with high concentrations of rosmarinic acid cannot grow on nutrient-rich plates [28]. It is consistent with the results of the study.

At present, commercially available antifungal drugs usually work by interfering with ergosterol synthesis, formation of water channels by ergosterol binding on cell membranes to interfere with cell wall synthesis and interference with nucleic acid replication [52]. However, the recent emergence of drug-resistant fungi in the world is mainly due to mutations in the squalene epoxidase gene and squalene epoxidase is a key link in the ergosterol synthesis pathway [7–9]. Therefore, there are significant implications in the antifungal field by developing a new pathway. As we all known, there was no report of antifungal drugs through affecting enolase expression. However, the proteomic results in this study indicated that the expression of enolase significantly decreased while *Trichophyton mentagrophytes* treated with rosmarinic acid (Fig. 9). There were studies shown that inhibition of enolase could have significant effects on cell growth [53]. Enolase is a key glycolytic metalloenzyme involved in carbon metabolism and is mainly responsible for the key step of catalyzing the conversion of 2-phosphoglycerate (2-PG) to phosphoenolpyruvate (PEP) in glycolysis, which provides substrate support for subsequent oxidative phosphorylation and is an important protein in glycolysis [54]. Most of the substrates they react to are carbohydrates or their derivatives, such as glycolic acid and amino acid [55]. In addition, the activation of the mitogen-activated protein kinase MAPK pathway could increase the expression of enolase [56]. The results of this study showed that there were 52 transcripts enriched in the MAPK pathway, and they were highly expressed in the rosmarinic acid treatment group (Fig. 8B). These results suggested rosmarinic acid could decrease the expression of enolase by blocking the upstream pathway of enolase synthesis. Related results were shown in Table S10 in Additional file 1.

Enolase serves as a prototype for metalloenzymes with labile metal ions that play a key role in catalytic turnover [57]. In the present study, the molecular docking results suggested that rosmarinic acid and the substrate-competitive inhibitor of enolase Hex and POMHEX could bind with the Magnesium ion in enolase via metal-acceptor interaction (Fig. 11). The reported studies have showed that the amino acid residues GLU211, LYS345, HIS159, GLU168 and ASP246 could be potential binding sites of the substrate 2-PG and enolase [57, 58]. Meanwhile, in the present study, the hydrogen bond interaction formed the rosmarinic acid and HIS159 residue. The bond of the GLU168, ASP246 and ASP323 residue with the benzene ring of rosmarinic acid was affected by Pi-anion and Pi-cation interaction. The hydrogen bond interaction formed the Hex and the GLU211 residue. And the bond of the HIS159 residue with the Hex and POMHEX were all affected by Pi-Alkyl interaction (Table 1). These results suggested that rosmarinic acid could be a substrate-competitive inhibitor of enolase.

## Conclusion

**Fig. 12:**
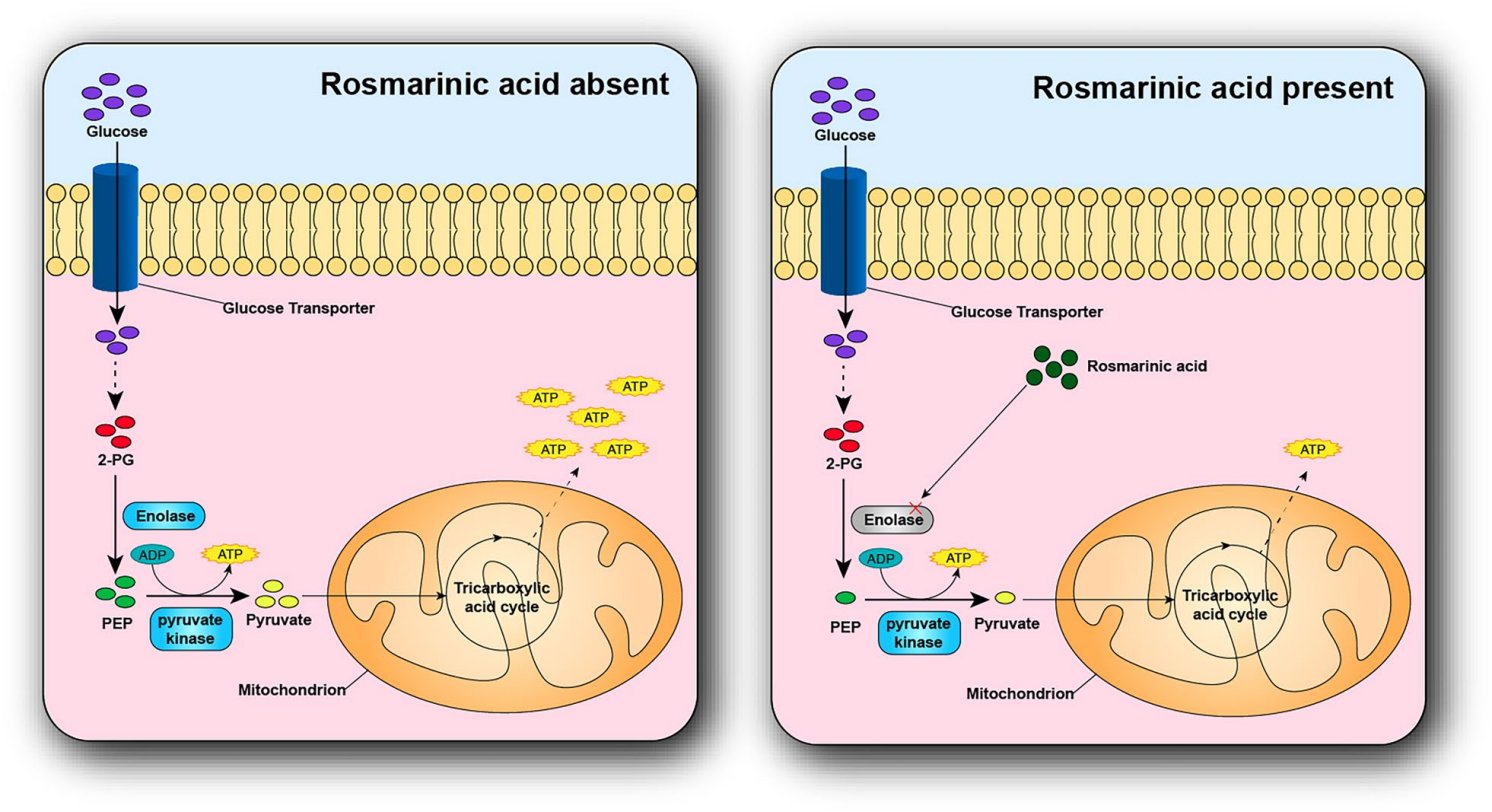
Diagram of possible inhibitory mechanisms of rosmarinic acid of *Trichophyton mentagrophytes* growth

The findings of this study demonstrated that rosmarinic acid has a certain degree of inhibition and lethal effect on the growth of *Trichophyton mentagrophytes*. And the preliminary mechanisms exploration indicated that the rosmarinic acid might affect the normal metabolism and glycolysis of *Trichophyton mentagrophytes* by inhibiting the expression of enolase, resulting in an insufficient intracellular supply of energy to fungi, thus inhibiting the growth of *Trichophyton mentagrophytes* due to insufficient energy supply for normal physiological activities (Fig. 12). This provided more extensive research ideas for the development of antifungal drugs.

## Supplementary Information

**Additional file 1: Method 1.** Library preparation for Transcriptome sequencing and transcriptomics data quality control and differential expression analysis. **Method 2.** Sample preparation of SDS-PAGE and data analysis. **Fig. S1.** Distribution of base sequencing error rates. **Fig. S2.** Distribution of ATCG content. **Fig. S3.** Muscari Unigene length Distribution **Fig. S4. **Comparative plot of the trimmed mean of M (TMM) density distribution. **Fig. S5. **Box line plot of TMM. **Table S1.** List of primer sequences for real-time PCR. **Table S2.** Active compounds of *P. frutescens*. **Table S3.** Diseases associated with Tiena and CTD ID. **Table S4.** The top five active compounds of *P. frutescens*. **Table S5.** Relationship between base score and error rate. **Table S6.** Sample sequencing data. **Table S7.** Assembly results. **Table S8.** Unigene annotated. **Table S9.** Differential genes. **Table S10.** Differential proteins.

## Data Availability

The datasets during and/or analyzed during the current study available from the corresponding author on reasonable request.

## Abbreviations

2-PG: 2-Phosphoglycerate
BCA: Bicinchoninic acid
BP: Biological process
CC: Cellular component
CLSI: Clinical and Laboratory Standards Institute
CTD: Comparative toxicogenomics database
DEGs: Differentially expressed genes
DL: Drug-likeness
DMSO: Dimethyl sulfoxide
FBS: Fetal bovine serum
FC: Fold change
FDR: False discovery rate
GO: Gene ontology
KEGG: Kyoko Encyclopedia of Genes and Genomes
MAPK: Mitogen-activated protein kinase
MBC: Minimum bactericidal concentration
MF: Molecular function
MIC: Minimum inhibitory concentration
PCR: Polymerase chain reaction
PDA: Potato dextrose agar
PEP: Phosphoenolpyruvate
PMSF: Phenylmethylsulfonyl fluoride
RIPA: Radio immunoprecipitation assay
RSEM: RNA-Seq by Expectation–Maximization
TCMSP: Traditional Chinese Medicine Systems Pharmacology Database and Analysis Platform
