# Supplementary material for: Integration of transcriptomics and proteomics to elucidate inhibitory effect and mechanism of rosmarinic acid from Perilla frutescens (L.) Britt. in treating Trichophyton mentagrophytes
Source: Chin Med. 2023 Jun 6;18:67. doi: 10.1186/s13020-023-00772-2 (PMC10245427; doi:10.1186/s13020-023-00772-2)
Supplement: Supplementary file 1 — Additional file 1: Method 1. Library preparation for Transcriptome sequencing and transcriptomics data quality control and differential expression analysis. Method 2. Sample preparation of SDS-PAGE and data analysis. Fig. S1. Distribution of base sequencing error rates. Fig. S2. Distribution of ATCG content. Fig. S3. Muscari Unigene length Distribution Fig. S4. Comparative plot of the trimmed mean of M (TMM) density distribution. Fig. S5. Box line plot of TMM. Table S1. List of primer sequences for real-time PCR. Table S2. Active compounds of P. frutescens. Table S3. Diseases associated with Tiena and CTD ID. Table S4. The top five active compounds of P. frutescens. Table S5. Relationship between base score and error rate. Table S6. Sample sequencing data. Table S7. Assembly results. Table S8. Unigene annotated. Table S9. Differential genes. Table S10. Differential proteins. [file 13020_2023_772_MOESM1_ESM.docx]

**Additional file 1**

**Additional file 1: Methods**

1. Library preparation for Transcriptome sequencing and transcriptomics data quality control and differential expression analysis.
2. Sample preparation of SDS-PAGE and data analysis.

**Additional file 1: Figures**

Figure 1. Distribution of base sequencing error rates.

Figure 2. Distribution of ATCG content.

Figure 3. Muscari Unigene length Distribution.

Figure 4. Comparative plot of the trimmed mean of M (TMM) density distribution.

Figure 5. Box line plot of TMM.

**Additional file 1: Tables**

Table 1. List of primer sequences for real-time PCR.

Table 2. Active compounds of *P. frutescens*.

Table 3. Diseases associated with Tiena and CTD ID.

Table 4. The top five active compounds of *P. frutescens*.

Table 5. Relationship between base score and error rate.

Table 6. Sample sequencing data.

Table 7. Assembly results.

Table 8. Unigene annotated.

Table 9. Differential genes.

Table 10. Differential proteins.

**Additional file 1: Methods**

1. Library preparation for Transcriptome sequencing and transcriptomics data quality control and differential expression analysis

RNA degradation and contamination were monitored on 1% agarose gels. RNA purity was checked using the NanoPhotometer^®^ spectrophotometer (IMPLEN, CA, USA). RNA concentration was measured using Qubit^®^ RNA Assay Kit in Qubit^®^2.0 Fluorometer (Life Technologies, CA, USA). RNA integrity was assessed using the RNA Nano 6000 Assay Kit of the Agilent Bioanalyzer 2100 system (Agilent Technologies, CA, USA).

A total amount of 3 μg RNA per sample was used as input material for the RNA sample preparations. Following the manufacturer's recommendations, sequencing libraries were generated using NEBNext®Ultra™ RNA Library Prep Kit for Illumina® (NEB, USA), and index codes were added to attribute sequences to each sample. Briefly, mRNA was purified from total RNA using poly-T oligo-attached magnetic beads. Fragmentation was performed using divalent cations under elevated temperature in NEBNext First Strand Synthesis Reaction Buffer (5X). First-strand cDNA was synthesized using random hexamer primer and M-MuLV Reverse Transcriptase (RNase H-). Second strand cDNA synthesis was performed using DNA Polymerase I and RNase H. Remaining overhangs were converted into blunt ends via exonuclease/polymerase activities. After adenylation of 3’ ends of DNA fragments, NEBNext Adaptor with hairpin loop structure was ligated to prepare for hybridization. To select cDNA fragments of preferentially 150~200 bp in length, the library fragments were purified with the AMPure XP system (Beckman Coulter, Beverly, USA). Then 3 μL USER Enzyme (NEB, USA) was used with size-selected, adaptor-ligated cDNA at 37 °C for 15 min, followed by 5 min at 95 °C before PCR. Then PCR was performed with Phusion High-Fidelity DNA polymerase, Universal PCR primers, and Index (X) Primer. At last, PCR products were purified (AMPure XP system), and library quality was assessed on the Agilent Bioanalyzer 2100 system.

According to the manufacturer's instructions, the index-coded samples were clustered on a cBot Cluster Generation System using TruSeq PE Cluster Kit v3-cBot-HS (Illumia). After cluster generation, the library preparations were sequenced on an Illumina Hiseq 2000 platform, and paired-end reads were generated. Raw data (raw reads) of fastq format were processed through in-house Perl scripts. In this step, clean data (clean reads) were obtained by removing reads containing adapter, ploy-N, and low-quality reads from raw data. At the same time, Q20, Q30, GC-content, and sequence duplication levels of the clean data were calculated. All the downstream analyses were based on clean data with high quality.

The left files (read1 files) from all libraries/samples were pooled into one big left.fq file, and right files (read2 files) into one big right.fq file. Transcriptome assembly was accomplished based on the left.fq and right.fq using Trinity with min_kmer_cov set to 2 by default and all other parameters set to default. RSEM estimated gene expression levels for each sample: clean data were mapped back onto the assembled transcriptome, and the read count for each gene was obtained from the mapping results. Differential expression analysis of two conditions/groups was performed using the DESeq R package (1.10.1). DESeq provides statistical routines for determining differential expression in digital gene expression data using a model based on the negative binomial distribution. The resulting P values were adjusted using Benjamini and Hochberg’s approach for controlling the false discovery rate. Genes with an adjusted P-value <0.05 found by DESeq were assigned as differentially expressed.

1. Sample preparation of SDS-PAGE and data analysis

The entire slab of the SDS-PAGE gel was rinsed twice with 1000 μL ddH_2_O for 1 hour. Bands (spots) of interest were excised with a clean scalpel and cut into cubes (about 1 mm); gel pieces were transferred into a new low-binding Eppendorf tube and spun down with microcentrifuge. Add 1000 μL decolorization solution to decolorize until the colloidal particles are colorless (replace the decolorization solution several times), and remove the decolorization solution. Gels were then incubated with 800 μL of neat CAN for 10 min until gel pieces shrunk (they became opaque and stuck together).

After removing all liquids, 600 μL of 5mM DTT solution (in 100 mM NH4HCO3) was added to cover gel pieces for 30 min at 55 ºC. Tubes chilled to room temperature, incubated with 800 μL of ACN for 10 min. For alkylation, 600 μL of 15 mM IAA solution (in 100 mM NH_4_HCO_3_) was incubated with gels for 40 min at room temperature in the dark. Alkylated protein gels were shrunk with ACN and collected with microcentrifuge. Trypsin digestion was performed by saturating the gels for 30 min with 600 μL trypsin (200 ng in 100 mM NH_4_CO_3_) buffer. Additional trypsin buffer covers the gels for 30 minutes, then incubates overnight at 37 ºC. Peptides were extracted with 800 μL of extraction buffer (1:2 (vol/vol) 5% formic acid/ACN) and vacuum dried. Peptides were LCMS analyzed.

For each sample, 5 μL of total peptides were separated and analyzed with a nano-UPLC (EASY-nLC1200) coupled to a Q Exactive HFX Orbitrap instrument (Thermo Fisher Scientific) with a nanoelectrospray ion source. Separation was performed using a reversed-phase column (100 μm ID × 15 cm, ReprosilPur 120 C18-AQ, 1.9 μm, Dr. Maisch). Mobile phases were H_2_O with 0.1% FA, 2% ACN (phase A) and 80% ACN, 0.1% FA (phase B). Separation of the sample was executed with a 60 min gradient at a 300 nL/min flow rate. Gradient B: 2-5% for 2 min, 5-22% for 44 min, 22-45% for 10 min, 45-95% for 2 min, 95% for 2 min.

Data-dependent acquisition (DDA) was performed in profile and positive mode with an Orbitrap analyzer at a resolution of 120,000 (@200 m/z) and m/z range of 350-1600 for MS1; For MS2, the resolution was set to 15,000 with a dynamic first mass. The automatic gain control (AGC) target for MS1 was set to 3E6 with max IT 50 ms and 1E5 for MS2 with max IT 110 ms. The top 20 most intense ions were fragmented by HCD with normalized collision energy (NCE) of 27% and an isolation window of 1.2 m/z. The dynamic exclusion time window was 45 s, and single charged peaks and peaks with charges exceeding 6 were excluded from the DDA procedure.

Vendor’s raw MS files were processed using Proteome Discoverer (PD) software (Version 2.4.0.305) and the built-in Sequest HT search engine. MS spectra lists were searched against their species-level UniPort FASTA databases (uniport-Trichophyton mentagrophytes 523103.fasta), Carbamidomethyl [C] as a fixed modification, Oxidation (M) and Acetyl (Protein N-term) as variable modifications. Trypsin was used as proteases. A maximum of 2 missed cleavage (s) was allowed. The false discovery rate (FDR) was set to 0.01 for both PSM and peptide levels. Peptide identification was performed with an initial precursor mass deviation of up to 10 ppm and a fragment mass deviation of 0.02 Da. Unique peptide and Razor peptide were used for protein quantification. All the other parameters were reserved as default.

**Additional file 1: Figures**


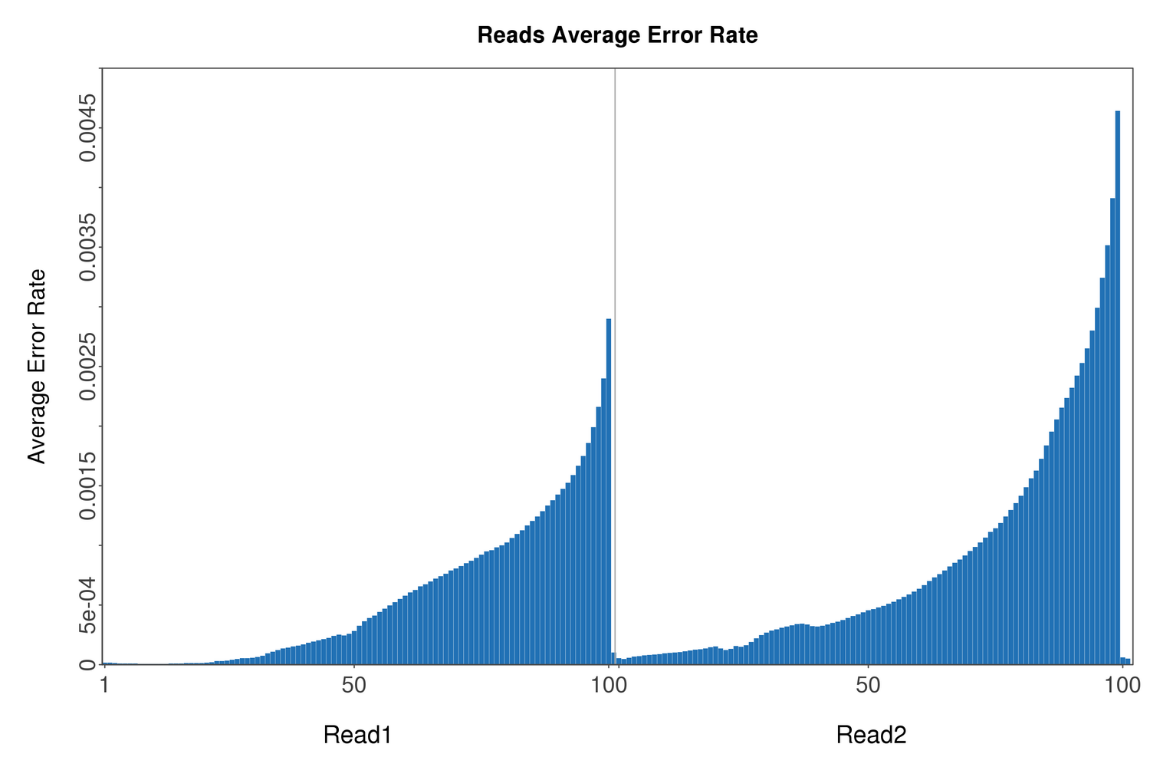


Figure 1. Distribution of base sequencing error rates. The horizontal coordinate is the base position of Reads, and the vertical coordinate is the single-base error rate.


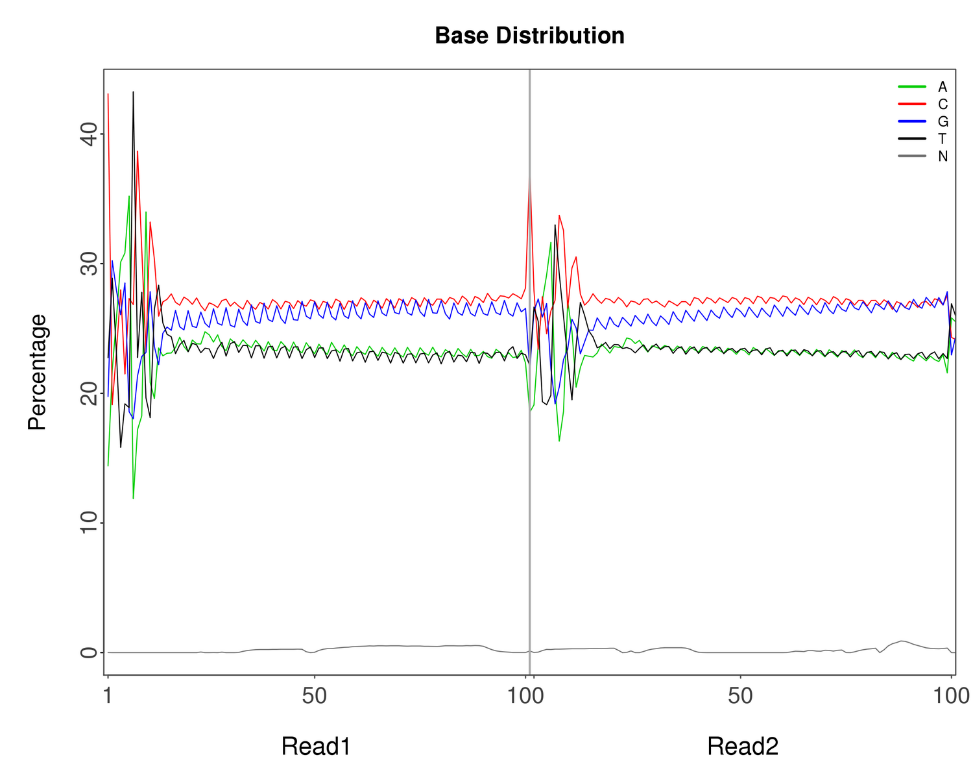


Figure 2. Distribution of ATCG content. The horizontal coordinates are the base positions of Reads, and the vertical coordinates are the proportion of single bases.


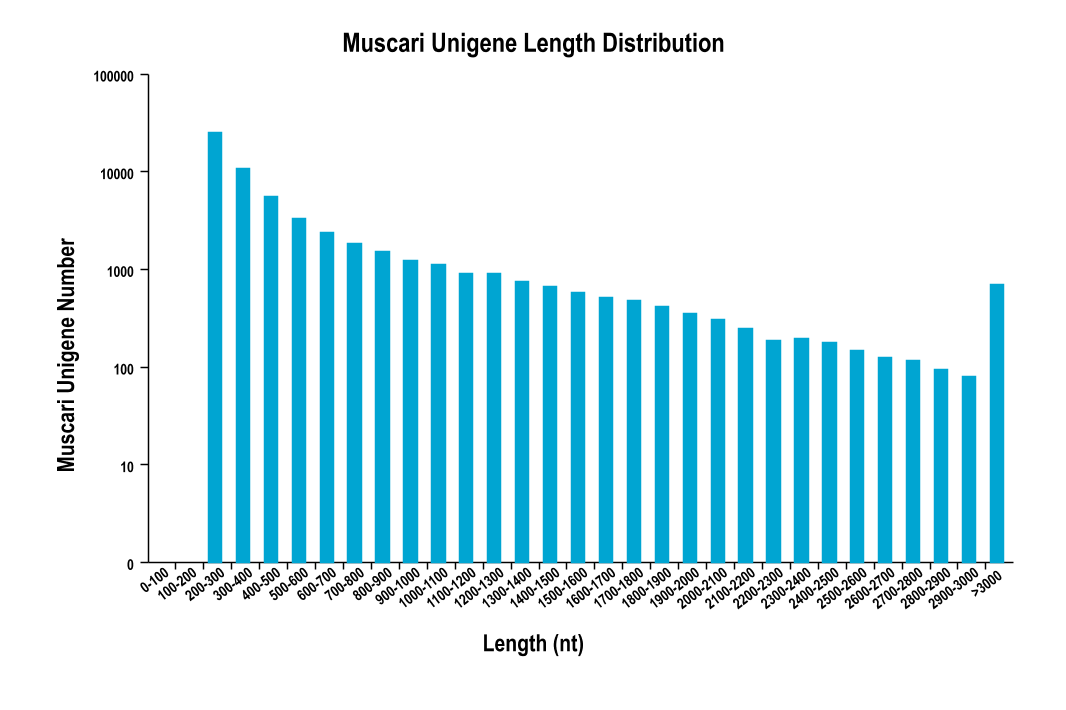


Figure 3. Muscari Unigene length Distribution. The horizontal coordinate is the transcript splicing length, and the vertical coordinate is the number of transcripts enriched to that long interval.


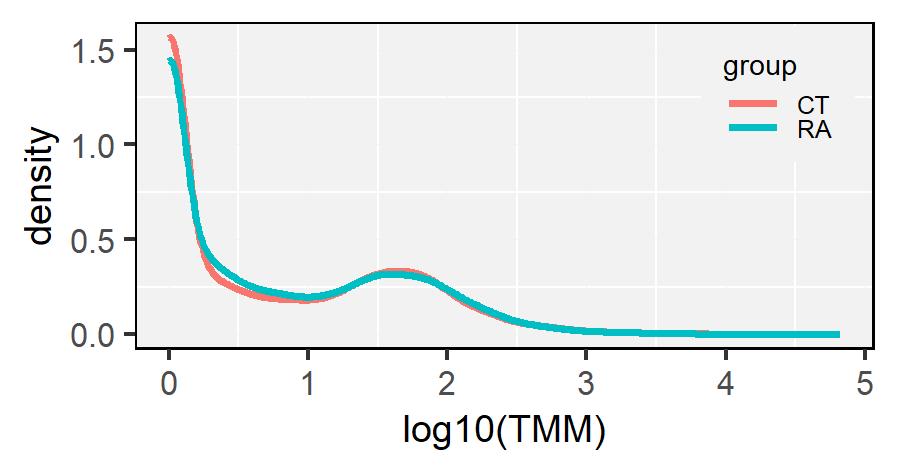


Figure 4. Comparative plot of TMM density distribution. The horizontal coordinates of the points on the curves indicate the logarithmic values of the TMM of the corresponding samples, and the vertical coordinates of the facts show the probability densities.


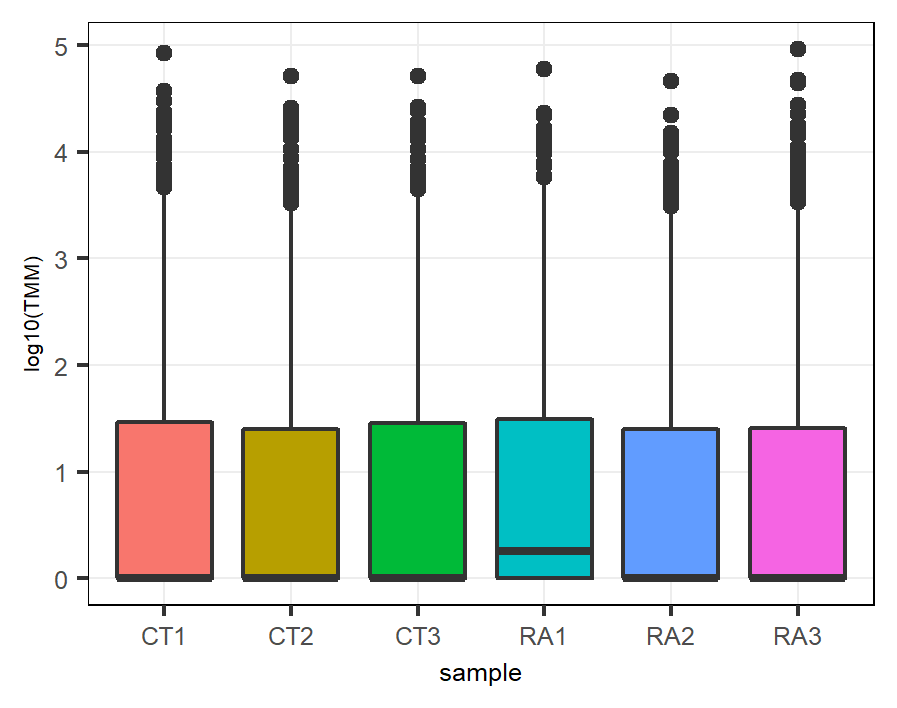


Figure 5. Box line plot of TMM. The horizontal coordinates represent the different samples; the vertical coordinates indicate the logarithmic values of the TMM of the sample expressions. The graph measures the expression level of each piece in terms of the overall expression dispersion.

**Additional file 1: Tables**

Table 1. List of primer sequences for real-time PCR

| Name | Forward primer (5'-3') | Reverse primer (5'-3') |
| --- | --- | --- |
| DN705_c0_g1_i2 | TCGGTTCTCGCTGTTTATTCTC | TTCACCTCCGTCCATCTACCAT |
| DN572_c3_g1_i1 | GTTCAGTCGGTTGGTCTGTGGT | GAGACACGCATTGGAGAACG |
| DN7_c4_g1_i8 | AAAGCACCCACCTCACTTCCAG | CCATTAGGTTGTGAGCCGTGA |
| DN19_c5_g4_i2 | ACGCACAAGCAACCGAGAAT | GGGCTTTGCCAGTGGTGATA |
| DN2177_c0_g1_i1 | GACTTCTGCTAATACCCGTTCAA | CGAAGATGGAGGTGATGGAAA |
| DN884_c0_g1_i4 | ATGTGCTGCCAGTTGGTTGATT | CACTTTGCCCACCTATTCATCC |
| DN494_c5_g1_i2 | TCTGCCTGACCTGTTACATCCC | CCGCAGTTACAAAGTTGGGTCT |
| 18s rRNA | AGGCGCGCAAATTACCCAATCC | GCCCTCCAATTGTTCCTCGTTAAG |

Table 2. Active compounds of *P. frutescens*.

| Number | Molecule ID | Molecule name | DL |
| --- | --- | --- | --- |
| PF1 | MOL000511 | ursolic acid | 0.75457 |
| PF2 | MOL002931 | scutellarin | 0.78574 |
| PF3 | MOL002737 | scutellarein | 0.24387 |
| PF4 | MOL011865 | rosmarinic acid | 0.35103 |
| PF5 | MOL006214 | progesterone | 0.43689 |
| PF6 | MOL001771 | poriferast-5-en-3beta-ol | 0.75034 |
| PF7 | MOL000263 | oleanolic acid | 0.75599 |
| PF8 | MOL000009 | luteolin-7-o-glucoside | 0.77998 |
| PF9 | MOL000006 | luteolin | 0.24552 |
| PF10 | MOL005030 | gondoic acid | 0.19743 |
| PF11 | MOL006210 | Eugenyl-β-D-glucopyranoside | 0.23285 |
| PF12 | MOL006209 | cyanin | 0.75918 |
| PF13 | MOL000002 | cyanidol | 0.24431 |
| PF14 | MOL000358 | beta-sitosterol | 0.75123 |
| PF15 | MOL002773 | beta-carotene | 0.58358 |
| PF16 | MOL000008 | apigenin | 0.21306 |
| PF17 | MOL005855 | Tormentic acid | 0.71209 |
| PF18 | MOL001747 | Tetracosane | 0.23973 |
| PF19 | MOL001402 | Octacosane | 0.36732 |
| PF20 | MOL007179 | Linolenic acid ethyl ester | 0.19694 |
| PF21 | MOL006202 | LAX | 0.20222 |
| PF22 | MOL000870 | HEXATRIACONTANE | 0.40911 |
| PF23 | MOL000007 | Cosmetin | 0.74097 |
| PF24 | MOL000953 | CLR | 0.67677 |
| PF25 | MOL000012 | Arachic acid | 0.19499 |
| PF26 | MOL006196 | 3-β-D-glucopyranosyloxy-5-phenylvaleric acid | 0.28098 |
| PF27 | MOL006194 | 3-β-D-glucopyranosyl-3-epi-2-isocucurbic acid | 0.31811 |
| PF28 | MOL006192 | (R, E)-3-(3-ethoxy-4-hydroxyphenyl)-2-((3-(3-ethoxy-4-hydroxyphenyl) acryloyl) oxy) propanoic acid | 0.4947 |
| PF29 | MOL000492 | (+)-catechin | 0.24164 |

Table 3. Diseases associated with Tiena and CTD ID.

| Number | Name | MeSH® ID |
| --- | --- | --- |
| D1 | Tinea Pedis | D014008 |
| D2 | Tinea Capitis | D014006 |
| D3 | Tinea | D014005 |
| D4 | Onychomycosis | D014009 |

Table 4. The top five active compounds of *P. frutescens*.

| Number | Molecule name | Betweenness centrality | Closeness centrality | Degree |
| --- | --- | --- | --- | --- |
| PF5 | progesterone | 0.083331 | 0.464789 | 43 |
| PF9 | luteolin | 0.056976 | 0.445946 | 37 |
| PF16 | apigenin | 0.056366 | 0.442953 | 36 |
| PF1 | ursolic acid | 0.033992 | 0.428571 | 31 |
| PF4 | rosmarinic acid | 0.014842 | 0.388235 | 16 |

Table 5. Relationship between base score and error rate.

| Q-score | Incorrect rate | Correct rate |
| --- | --- | --- |
| Q10 | 1/10 | 90% |
| Q20 | 1/100 | 99% |
| Q30 | 1/1000 | 99.9% |
| Q40 | 1/10000 | 99.99% |

Table 6. Sample sequencing data.

| Samples | Read Number | Base Number | GC Content | % ≥ Q30 |
| --- | --- | --- | --- | --- |
| CT1 | 40,557,150 | 6,067,303,000 | 52.52% | 92.02% |
| CT2 | 41,334,450 | 6,186,365,000 | 52.67% | 93.52% |
| CT3 | 37,804,000 | 5,657,608,000 | 52.70% | 92.68% |
| RA1 | 43,404,224 | 6,494,997,000 | 52.35% | 92.78% |
| RA2 | 39,284,448 | 5,878,260,000 | 52.44% | 92.70% |
| RA3 | 40,773,516 | 6,100,232,000 | 52.71% | 93.22% |

Table 7. Assembly results.

| Length Range | Transcript | Unigene |
| --- | --- | --- |
| 200-300 | 5,796(15.25%) | 5,373(33.69%) |
| 300-500 | 3,991(10.50%) | 3,383(21.22%) |
| 500-1000 | 3,099(8.15%) | 1,979(12.41%) |
| 1000-2000 | 3,849(10.13%) | 1,452(9.11%) |
| 2000-3000 | 3,556(9.36%) | 1,016(6.37%) |
| 3000+ | 17,715(46.61%) | 2,743(17.20%) |
| Total Number | 38,006 | 15,946 |
| N50 Length | 2,627 | 431 |
| Mean Length | 3702 | 1552 |

Table 8. Unigene annotated.

| Anno_Database | Annotated_Number | 300 ≤ length < 1000 | Length ≥ 1000 |
| --- | --- | --- | --- |
| COG_Annotation | 13,237 | 4,289 | 3,978 |
| GO_Annotation | 12,977 | 4,152 | 3,868 |
| KEGG_Annotation | 9,425 | 2,515 | 1,985 |
| Pfam_Annotation | 9,147 | 3,222 | 3,009 |
| eggNOG_Annotation | 13,237 | 4,289 | 3,978 |

Table 9. Differential genes.

| No. | NCBI Reference Sequence | query | logFC | P-value | FDR |
| --- | --- | --- | --- | --- | --- |
| 1 | PROSTU_00109 | TRINITY_DN1598_c0_g1_i1 | 7.605147 | 1.25E-04 | 2.95E-03 |
| 2 | F2PQK9 | TRINITY_DN677_c2_g2_i7 | 3.095833 | 8.41E-28 | 4.45E-24 |
| 3 | F2PJZ1 | TRINITY_DN2177_c0_g1_i1 | 2.685322 | 6.26E-16 | 5.52E-13 |
| 4 | F2PUA2 | TRINITY_DN1548_c0_g1_i2 | 2.644411 | 5.27E-11 | 1.74E-08 |
| 5 | NA | TRINITY_DN576_c1_g3_i2 | 2.420688 | 3.70E-04 | 6.63E-03 |
| 6 | XP_003015688.1 | TRINITY_DN83_c11_g1_i1 | 2.215897 | 5.87E-07 | 4.64E-05 |
| 7 | F2PKD3 | TRINITY_DN1186_c2_g2_i1 | 2.160839 | 1.86E-03 | 2.12E-02 |
| 8 | F2PT77 | TRINITY_DN83_c42_g1_i1 | 2.150284 | 8.35E-05 | 2.08E-03 |
| 9 | XP_003017238.1 | TRINITY_DN1637_c0_g1_i3 | 2.132097 | 1.80E-12 | 6.34E-10 |
| 10 | F2Q1P9 | TRINITY_DN22043_c0_g1_i1 | 2.100019 | 3.53E-03 | 3.49E-02 |
| 11 | F2Q1M7 | TRINITY_DN1615_c0_g1_i1 | 1.975788 | 1.61E-04 | 3.41E-03 |
| 12 | XP_003014022.1 | TRINITY_DN984_c2_g1_i2 | 1.890272 | 6.38E-04 | 1.01E-02 |
| 13 | F2PI13 | TRINITY_DN1320_c0_g1_i1 | 1.857569 | 2.66E-05 | 9.33E-04 |
| 14 | F2Q0J1 | TRINITY_DN79_c4_g2_i2 | 1.812861 | 9.62E-09 | 1.59E-06 |
| 15 | XP_003015000.1 | TRINITY_DN282_c0_g2_i3 | 1.80672 | 1.62E-09 | 3.19E-07 |
| 16 | NA | TRINITY_DN656_c1_g1_i7 | 1.804724 | 1.17E-09 | 2.62E-07 |
| 17 | F2PS48 | TRINITY_DN43_c0_g1_i1 | 1.715461 | 1.52E-13 | 8.07E-11 |
| 18 | F2PV27 | TRINITY_DN7_c12_g1_i9 | 1.67369 | 1.44E-10 | 4.01E-08 |
| 19 | F2Q2B9 | TRINITY_DN656_c1_g2_i8 | 1.620062 | 2.62E-07 | 2.43E-05 |
| 20 | NA | TRINITY_DN622_c9_g1_i2 | 1.577106 | 5.41E-03 | 4.65E-02 |
| 21 | XP_003234268.1 | TRINITY_DN1709_c0_g1_i1 | 1.467341 | 1.05E-03 | 1.43E-02 |
| 22 | NA | TRINITY_DN272_c1_g2_i2 | 1.465983 | 2.92E-03 | 2.96E-02 |
| 23 | NA | TRINITY_DN265_c0_g10_i1 | 1.456469 | 4.28E-03 | 3.99E-02 |
| 24 | F2PX69 | TRINITY_DN18_c1_g2_i1 | 1.427192 | 2.61E-05 | 9.22E-04 |
| 25 | F2PI85 | TRINITY_DN1606_c0_g1_i1 | 1.406781 | 1.46E-05 | 5.75E-04 |
| 26 | F2PU60 | TRINITY_DN1868_c0_g1_i3 | 1.405805 | 1.43E-03 | 1.75E-02 |
| 27 | F2PZ40 | TRINITY_DN680_c0_g1_i1 | 1.387081 | 3.97E-06 | 2.08E-04 |
| 28 | F2Q456 | TRINITY_DN487_c1_g2_i2 | 1.38425 | 1.72E-03 | 2.00E-02 |
| 29 | F2PGV0 | TRINITY_DN161_c0_g1_i16 | 1.370886 | 2.75E-05 | 9.43E-04 |
| 30 | F2PRA6 | TRINITY_DN1806_c0_g1_i1 | 1.367709 | 8.21E-08 | 1.03E-05 |
| 31 | F2Q595 | TRINITY_DN1422_c1_g1_i4 | 1.337491 | 2.88E-07 | 2.59E-05 |
| 32 | F2PWQ8 | TRINITY_DN1247_c2_g1_i1 | 1.335982 | 9.03E-11 | 2.81E-08 |
| 33 | XP_003236032.1 | TRINITY_DN22089_c0_g1_i1 | 1.32545 | 5.15E-03 | 4.50E-02 |
| 34 | F2PTQ7 | TRINITY_DN178_c1_g1_i3 | 1.321389 | 8.33E-07 | 6.12E-05 |
| 35 | F2PW36 | TRINITY_DN54_c0_g2_i1 | 1.320037 | 2.99E-04 | 5.61E-03 |
| 36 | F2PZV7 | TRINITY_DN998_c1_g1_i2 | 1.318593 | 9.10E-07 | 6.59E-05 |
| 37 | F2PMW0 | TRINITY_DN414_c0_g2_i4 | 1.316444 | 1.04E-04 | 2.54E-03 |
| 38 | XP_003011711.1 | TRINITY_DN1174_c0_g1_i2 | 1.299226 | 4.34E-03 | 4.00E-02 |
| 39 | XP_003014058.1 | TRINITY_DN265_c9_g1_i2 | 1.293855 | 2.01E-04 | 4.10E-03 |
| 40 | F2PZ60 | TRINITY_DN1719_c0_g2_i1 | 1.28712 | 1.40E-06 | 8.63E-05 |
| 41 | F2PRC3 | TRINITY_DN941_c0_g1_i3 | 1.273292 | 1.57E-04 | 3.39E-03 |
| 42 | F2PVW2 | TRINITY_DN1849_c0_g2_i1 | 1.261236 | 5.49E-05 | 1.51E-03 |
| 43 | XP_003231889.1 | TRINITY_DN323_c1_g1_i22 | 1.24917 | 9.64E-08 | 1.09E-05 |
| 44 | F2PLA0 | TRINITY_DN18946_c0_g1_i1 | 1.246783 | 1.44E-04 | 3.20E-03 |
| 45 | XP_003011306.1 | TRINITY_DN175_c4_g3_i3 | 1.236428 | 5.49E-03 | 4.66E-02 |
| 46 | XP_007801075.1 | TRINITY_DN460_c2_g2_i2 | 1.230767 | 1.47E-03 | 1.79E-02 |
| 47 | XP_003011304.1 | TRINITY_DN647_c0_g1_i1 | 1.222622 | 1.03E-06 | 7.24E-05 |
| 48 | XP_003238615.1 | TRINITY_DN1198_c0_g1_i1 | 1.219322 | 6.14E-04 | 9.79E-03 |
| 49 | XP_003237467.1 | TRINITY_DN1615_c0_g2_i3 | 1.211382 | 5.49E-03 | 4.66E-02 |
| 50 | F2PJ82 | TRINITY_DN387_c0_g1_i5 | 1.206486 | 1.86E-04 | 3.85E-03 |
| 51 | F2PNN1 | TRINITY_DN128_c0_g1_i3 | 1.204886 | 6.84E-05 | 1.81E-03 |
| 52 | XP_003012969.1 | TRINITY_DN127_c1_g1_i2 | 1.201248 | 6.30E-04 | 9.98E-03 |
| 53 | XP_003231283.1 | TRINITY_DN494_c5_g1_i2 | 1.201067 | 1.15E-04 | 2.75E-03 |
| 54 | F2Q4Q0 | TRINITY_DN317_c1_g2_i1 | 1.172217 | 1.28E-03 | 1.64E-02 |
| 55 | XP_003236094.1 | TRINITY_DN412_c0_g1_i1 | 1.169663 | 2.48E-04 | 4.82E-03 |
| 56 | F2PNJ7 | TRINITY_DN815_c1_g1_i1 | 1.165379 | 1.63E-05 | 6.29E-04 |
| 57 | F2PVD6 | TRINITY_DN1454_c0_g1_i3 | 1.162565 | 9.10E-08 | 1.07E-05 |
| 58 | F2Q1W4 | TRINITY_DN1268_c0_g1_i2 | 1.149825 | 2.42E-03 | 2.57E-02 |
| 59 | F2PPW3 | TRINITY_DN844_c0_g1_i2 | 1.148467 | 4.42E-04 | 7.60E-03 |
| 60 | F2Q5J5 | TRINITY_DN928_c1_g2_i1 | 1.147673 | 8.99E-04 | 1.27E-02 |
| 61 | XP_003013485.1 | TRINITY_DN1023_c3_g1_i1 | 1.146022 | 1.79E-03 | 2.06E-02 |
| 62 | F2PQV0 | TRINITY_DN7_c19_g2_i1 | 1.133291 | 5.29E-06 | 2.50E-04 |
| 63 | F2PLY7 | TRINITY_DN101_c0_g2_i1 | 1.125516 | 1.19E-09 | 2.62E-07 |
| 64 | F2PPZ6 | TRINITY_DN5_c7_g1_i1 | 1.123557 | 3.57E-09 | 6.75E-07 |
| 65 | F2PJE5 | TRINITY_DN72_c0_g1_i70 | 1.117134 | 1.92E-03 | 2.16E-02 |
| 66 | XP_003236465.1 | TRINITY_DN152_c6_g1_i1 | 1.116068 | 1.49E-05 | 5.84E-04 |
| 67 | F2PX73 | TRINITY_DN265_c2_g1_i1 | 1.106377 | 6.41E-09 | 1.09E-06 |
| 68 | XP_003013787.1 | TRINITY_DN1328_c0_g2_i4 | 1.105746 | 4.72E-03 | 4.27E-02 |
| 69 | F2PZZ1 | TRINITY_DN1710_c0_g1_i2 | 1.099985 | 1.63E-09 | 3.19E-07 |
| 70 | XP_003232700.1 | TRINITY_DN1858_c0_g1_i2 | 1.09947 | 8.14E-08 | 1.03E-05 |
| 71 | F2PKZ8 | TRINITY_DN1125_c3_g1_i6 | 1.09845 | 5.98E-04 | 9.65E-03 |
| 72 | XP_003012640.1 | TRINITY_DN884_c0_g1_i4 | 1.097082 | 2.46E-03 | 2.59E-02 |
| 73 | XP_003015430.1 | TRINITY_DN2019_c0_g1_i2 | 1.088719 | 1.13E-06 | 7.47E-05 |
| 74 | XP_003011738.1 | TRINITY_DN2282_c0_g1_i1 | 1.08865 | 7.53E-05 | 1.91E-03 |
| 75 | F2PWA1 | TRINITY_DN1908_c0_g1_i3 | 1.081921 | 1.35E-03 | 1.69E-02 |
| 76 | F2Q5Y6 | TRINITY_DN2_c0_g4_i1 | 1.075803 | 1.47E-04 | 3.25E-03 |
| 77 | XP_003232249.1 | TRINITY_DN186_c12_g1_i1 | 1.074536 | 2.64E-04 | 5.10E-03 |
| 78 | XP_003011197.1 | TRINITY_DN1849_c3_g1_i4 | 1.071746 | 4.56E-06 | 2.30E-04 |
| 79 | XP_003009955.1 | TRINITY_DN2033_c0_g1_i2 | 1.069501 | 1.15E-07 | 1.26E-05 |
| 80 | F2PQU8 | TRINITY_DN1373_c0_g1_i1 | 1.066989 | 3.02E-06 | 1.70E-04 |
| 81 | F2PRR8 | TRINITY_DN743_c0_g1_i2 | 1.058693 | 1.24E-03 | 1.61E-02 |
| 82 | XP_003016932.1 | TRINITY_DN1378_c0_g2_i1 | 1.052232 | 4.72E-05 | 1.36E-03 |
| 83 | F2PQJ2 | TRINITY_DN359_c1_g1_i1 | 1.051507 | 5.26E-05 | 1.48E-03 |
| 84 | F2PI31 | TRINITY_DN139_c1_g1_i12 | 1.050297 | 1.74E-04 | 3.64E-03 |
| 85 | F2Q1U9 | TRINITY_DN1758_c0_g2_i1 | 1.049033 | 2.02E-05 | 7.44E-04 |
| 86 | NA | TRINITY_DN635_c0_g3_i2 | 1.042063 | 4.95E-03 | 4.41E-02 |
| 87 | F2PLU4 | TRINITY_DN271_c1_g1_i2 | 1.041249 | 1.17E-06 | 7.57E-05 |
| 88 | XP_003012446.1 | TRINITY_DN223_c1_g1_i2 | 1.04098 | 4.46E-05 | 1.31E-03 |
| 89 | F2PIG2 | TRINITY_DN1488_c0_g1_i2 | 1.040078 | 7.34E-05 | 1.89E-03 |
| 90 | F2Q2T9 | TRINITY_DN1180_c2_g1_i1 | 1.038988 | 1.63E-03 | 1.93E-02 |
| 91 | F2PII8 | TRINITY_DN800_c1_g1_i2 | 1.036142 | 3.71E-04 | 6.63E-03 |
| 92 | XP_003015364.1 | TRINITY_DN427_c0_g1_i6 | 1.035025 | 1.86E-04 | 3.85E-03 |
| 93 | F2Q2G2 | TRINITY_DN150_c1_g1_i1 | 1.031467 | 2.75E-05 | 9.43E-04 |
| 94 | F2PHG3 | TRINITY_DN1813_c0_g1_i1 | 1.029619 | 4.69E-03 | 4.25E-02 |
| 95 | XP_003010567.1 | TRINITY_DN1142_c0_g1_i1 | 1.022996 | 6.32E-05 | 1.69E-03 |
| 96 | F2PRD7 | TRINITY_DN1639_c0_g1_i4 | 1.022823 | 1.13E-05 | 4.58E-04 |
| 97 | F2PT54 | TRINITY_DN1051_c0_g1_i1 | 1.022125 | 1.41E-04 | 3.20E-03 |
| 98 | F2Q2Y5 | TRINITY_DN381_c9_g1_i1 | 1.02157 | 4.65E-03 | 4.22E-02 |
| 99 | F2PZL3 | TRINITY_DN1133_c3_g2_i4 | 1.020248 | 2.49E-08 | 3.56E-06 |
| 100 | F2PRJ7 | TRINITY_DN664_c1_g1_i1 | 1.014803 | 6.30E-04 | 9.98E-03 |
| 101 | F2PI37 | TRINITY_DN1462_c0_g1_i3 | 1.014311 | 5.06E-03 | 4.44E-02 |
| 102 | F2PHM0 | TRINITY_DN3082_c0_g1_i1 | 1.012622 | 9.10E-06 | 3.85E-04 |
| 103 | NA | TRINITY_DN18_c1_g3_i1 | 1.010117 | 2.24E-03 | 2.44E-02 |
| 104 | F2PP87 | TRINITY_DN636_c1_g1_i2 | 1.004226 | 3.46E-05 | 1.10E-03 |
| 105 | F2PJR3 | TRINITY_DN20_c3_g1_i16 | 1.003454 | 5.29E-03 | 4.58E-02 |
| 106 | F2PXR5 | TRINITY_DN336_c1_g2_i9 | -1.00023 | 1.97E-04 | 4.05E-03 |
| 107 | F2Q1J7 | TRINITY_DN1547_c4_g1_i1 | -1.00268 | 5.63E-04 | 9.13E-03 |
| 108 | F2PUY3 | TRINITY_DN21256_c0_g1_i1 | -1.00285 | 6.29E-05 | 1.69E-03 |
| 109 | F2PML8 | TRINITY_DN121_c1_g1_i8 | -1.00359 | 3.39E-08 | 4.72E-06 |
| 110 | XP_003014949.1 | TRINITY_DN1038_c1_g1_i2 | -1.00786 | 2.83E-06 | 1.61E-04 |
| 111 | F2PUV6 | TRINITY_DN325_c0_g1_i3 | -1.01338 | 8.71E-08 | 1.07E-05 |
| 112 | NA | TRINITY_DN1597_c1_g1_i1 | -1.01867 | 1.42E-04 | 3.20E-03 |
| 113 | F2Q282 | TRINITY_DN826_c0_g1_i1 | -1.02071 | 1.04E-04 | 2.54E-03 |
| 114 | XP_003238357.1 | TRINITY_DN36_c5_g2_i2 | -1.02169 | 3.07E-05 | 1.01E-03 |
| 115 | F2Q499 | TRINITY_DN261_c2_g1_i36 | -1.02729 | 2.26E-04 | 4.51E-03 |
| 116 | F2PLA4 | TRINITY_DN1021_c0_g1_i2 | -1.02959 | 3.62E-05 | 1.13E-03 |
| 117 | XP_003011982.1 | TRINITY_DN622_c0_g1_i28 | -1.03156 | 1.29E-03 | 1.64E-02 |
| 118 | XP_003014943.1 | TRINITY_DN439_c0_g3_i2 | -1.03487 | 2.98E-07 | 2.63E-05 |
| 119 | F2PZN1 | TRINITY_DN1000_c0_g1_i11 | -1.03581 | 7.99E-05 | 2.00E-03 |
| 120 | F2PP24 | TRINITY_DN1555_c0_g1_i1 | -1.03634 | 9.25E-04 | 1.29E-02 |
| 121 | F2PQC9 | TRINITY_DN2047_c0_g1_i1 | -1.03857 | 1.13E-03 | 1.51E-02 |
| 122 | F2PRQ9 | TRINITY_DN719_c0_g1_i14 | -1.04951 | 3.57E-06 | 1.93E-04 |
| 123 | F2PZI4 | TRINITY_DN222_c0_g9_i2 | -1.05143 | 7.12E-04 | 1.10E-02 |
| 124 | F2PJD7 | TRINITY_DN19_c14_g1_i3 | -1.05326 | 3.98E-03 | 3.82E-02 |
| 125 | F2PIN5 | TRINITY_DN1683_c0_g1_i7 | -1.05483 | 4.09E-03 | 3.87E-02 |
| 126 | F2PRB6 | TRINITY_DN439_c0_g2_i2 | -1.057 | 1.67E-04 | 3.52E-03 |
| 127 | F2Q0F4 | TRINITY_DN993_c0_g1_i3 | -1.06009 | 1.05E-06 | 7.24E-05 |
| 128 | F2PHR7 | TRINITY_DN558_c0_g1_i7 | -1.06278 | 5.13E-05 | 1.45E-03 |
| 129 | XP_003238463.1 | TRINITY_DN778_c0_g1_i9 | -1.06776 | 1.07E-04 | 2.59E-03 |
| 130 | F2PXI6 | TRINITY_DN161_c5_g1_i1 | -1.07596 | 1.72E-07 | 1.76E-05 |
| 131 | F2PTT7 | TRINITY_DN175_c2_g1_i1 | -1.08182 | 3.14E-04 | 5.81E-03 |
| 132 | XP_003015158.1 | TRINITY_DN890_c0_g1_i4 | -1.0824 | 6.44E-06 | 2.94E-04 |
| 133 | NA | TRINITY_DN447_c3_g4_i2 | -1.08713 | 1.59E-04 | 3.40E-03 |
| 134 | F2PHJ5 | TRINITY_DN764_c1_g1_i7 | -1.08921 | 3.12E-04 | 5.80E-03 |
| 135 | XP_003238640.1 | TRINITY_DN153_c1_g1_i4 | -1.09105 | 5.74E-06 | 2.66E-04 |
| 136 | F2Q251 | TRINITY_DN1443_c1_g1_i1 | -1.09301 | 9.34E-04 | 1.30E-02 |
| 137 | F2PWL7 | TRINITY_DN196_c2_g2_i1 | -1.09538 | 5.18E-04 | 8.63E-03 |
| 138 | NA | TRINITY_DN297_c0_g1_i1 | -1.09892 | 7.37E-05 | 1.89E-03 |
| 139 | F2PKK2 | TRINITY_DN296_c0_g2_i1 | -1.10066 | 7.43E-07 | 5.69E-05 |
| 140 | F2PHU8 | TRINITY_DN1009_c0_g1_i2 | -1.10693 | 1.43E-04 | 3.20E-03 |
| 141 | F2PZE3 | TRINITY_DN380_c1_g1_i3 | -1.11643 | 1.06E-06 | 7.24E-05 |
| 142 | F2PIM9 | TRINITY_DN978_c0_g2_i1 | -1.11659 | 1.27E-04 | 2.99E-03 |
| 143 | F2Q0U2 | TRINITY_DN189_c7_g3_i6 | -1.12694 | 6.31E-07 | 4.91E-05 |
| 144 | NA | TRINITY_DN66_c13_g1_i5 | -1.13135 | 1.32E-03 | 1.66E-02 |
| 145 | F2Q095 | TRINITY_DN1104_c2_g1_i2 | -1.14225 | 4.34E-07 | 3.70E-05 |
| 146 | F2Q1H4 | TRINITY_DN6234_c0_g1_i1 | -1.14817 | 8.33E-04 | 1.23E-02 |
| 147 | XP_003232516.1 | TRINITY_DN0_c8_g1_i2 | -1.15423 | 6.70E-04 | 1.05E-02 |
| 148 | NA | TRINITY_DN19_c1_g3_i1 | -1.1594 | 1.49E-03 | 1.80E-02 |
| 149 | F2Q133 | TRINITY_DN1587_c0_g1_i1 | -1.16451 | 7.35E-06 | 3.30E-04 |
| 150 | F2Q3Q4 | TRINITY_DN622_c8_g1_i1 | -1.1684 | 1.97E-03 | 2.21E-02 |
| 151 | NA | TRINITY_DN317_c12_g1_i2 | -1.18148 | 2.68E-03 | 2.78E-02 |
| 152 | F2PLM2 | TRINITY_DN733_c0_g1_i6 | -1.18319 | 2.51E-05 | 8.92E-04 |
| 153 | NA | TRINITY_DN93_c1_g2_i4 | -1.18832 | 4.34E-03 | 4.00E-02 |
| 154 | NA | TRINITY_DN574_c2_g1_i4 | -1.18937 | 2.68E-03 | 2.78E-02 |
| 155 | F2PX83 | TRINITY_DN44_c6_g1_i2 | -1.19692 | 4.56E-05 | 1.33E-03 |
| 156 | NA | TRINITY_DN61_c4_g1_i2 | -1.198 | 1.51E-04 | 3.31E-03 |
| 157 | F2PV15 | TRINITY_DN66_c1_g2_i1 | -1.20567 | 3.27E-05 | 1.05E-03 |
| 158 | XP_003013841.1 | TRINITY_DN923_c0_g1_i3 | -1.20682 | 1.14E-04 | 2.73E-03 |
| 159 | F2PX37 | TRINITY_DN1995_c0_g1_i1 | -1.20891 | 8.25E-04 | 1.23E-02 |
| 160 | XP_003013408.1 | TRINITY_DN1712_c1_g1_i1 | -1.21131 | 8.68E-06 | 3.73E-04 |
| 161 | F2PHJ3 | TRINITY_DN764_c0_g1_i2 | -1.2182 | 5.07E-09 | 9.24E-07 |
| 162 | F2PPK1 | TRINITY_DN485_c0_g1_i1 | -1.22359 | 3.57E-05 | 1.12E-03 |
| 163 | F2Q4M7 | TRINITY_DN425_c1_g3_i5 | -1.23629 | 1.65E-05 | 6.34E-04 |
| 164 | XP_003017356.1 | TRINITY_DN222_c0_g7_i1 | -1.2368 | 4.91E-06 | 2.43E-04 |
| 165 | XP_003234356.1 | TRINITY_DN254_c4_g1_i4 | -1.23828 | 7.38E-10 | 1.78E-07 |
| 166 | XP_003015543.1 | TRINITY_DN1588_c0_g1_i2 | -1.24238 | 3.32E-06 | 1.83E-04 |
| 167 | XP_003010204.1 | TRINITY_DN1175_c0_g1_i3 | -1.2537 | 1.62E-05 | 6.29E-04 |
| 168 | F2Q260 | TRINITY_DN263_c0_g2_i19 | -1.25627 | 1.95E-04 | 4.01E-03 |
| 169 | NA | TRINITY_DN34_c2_g2_i1 | -1.26408 | 3.86E-06 | 2.06E-04 |
| 170 | XP_003238965.1 | TRINITY_DN697_c2_g2_i5 | -1.26962 | 2.02E-07 | 1.94E-05 |
| 171 | XP_003010572.1 | TRINITY_DN857_c2_g2_i3 | -1.27797 | 5.00E-07 | 4.11E-05 |
| 172 | F2PWQ1 | TRINITY_DN849_c3_g1_i3 | -1.29072 | 2.25E-08 | 3.31E-06 |
| 173 | F2PKF5 | TRINITY_DN229_c0_g1_i6 | -1.29777 | 1.25E-06 | 7.90E-05 |
| 174 | XP_003231743.1 | TRINITY_DN1680_c0_g1_i5 | -1.30078 | 1.04E-10 | 3.06E-08 |
| 175 | F2Q1C7 | TRINITY_DN839_c0_g1_i2 | -1.31931 | 9.35E-06 | 3.93E-04 |
| 176 | F2PT88 | TRINITY_DN261_c1_g3_i2 | -1.32195 | 5.11E-04 | 8.55E-03 |
| 177 | F2PJ90 | TRINITY_DN884_c6_g1_i1 | -1.32915 | 1.53E-03 | 1.84E-02 |
| 178 | NA | TRINITY_DN256_c2_g1_i12 | -1.33901 | 6.31E-05 | 1.69E-03 |
| 179 | F2PT63 | TRINITY_DN388_c0_g1_i29 | -1.34324 | 5.05E-06 | 2.43E-04 |
| 180 | XP_003013528.1 | TRINITY_DN14_c0_g1_i15 | -1.34868 | 2.00E-07 | 1.94E-05 |
| 181 | NA | TRINITY_DN11830_c0_g4_i1 | -1.35008 | 4.09E-05 | 1.24E-03 |
| 182 | F2PS97 | TRINITY_DN195_c1_g1_i6 | -1.35045 | 3.52E-07 | 3.05E-05 |
| 183 | F2PWN8 | TRINITY_DN2281_c0_g1_i1 | -1.35861 | 1.70E-03 | 1.99E-02 |
| 184 | NA | TRINITY_DN19443_c0_g1_i1 | -1.35881 | 3.04E-05 | 1.01E-03 |
| 185 | NA | TRINITY_DN118_c1_g1_i2 | -1.36538 | 1.24E-03 | 1.61E-02 |
| 186 | XP_003231168.1 | TRINITY_DN1225_c2_g1_i3 | -1.36617 | 1.03E-04 | 2.53E-03 |
| 187 | XP_003238427.1 | TRINITY_DN511_c6_g1_i1 | -1.37095 | 1.68E-08 | 2.54E-06 |
| 188 | XP_003238208.1 | TRINITY_DN1369_c1_g1_i2 | -1.37513 | 2.71E-04 | 5.21E-03 |
| 189 | F2PZT2 | TRINITY_DN685_c0_g1_i1 | -1.38055 | 1.40E-05 | 5.59E-04 |
| 190 | XP_003238360.1 | TRINITY_DN36_c2_g2_i6 | -1.38418 | 2.15E-06 | 1.30E-04 |
| 191 | F2Q0W6 | TRINITY_DN3286_c0_g1_i1 | -1.3969 | 6.22E-10 | 1.57E-07 |
| 192 | F2PGV7 | TRINITY_DN157_c5_g1_i3 | -1.41955 | 4.22E-05 | 1.26E-03 |
| 193 | F2Q5D1 | TRINITY_DN716_c1_g1_i1 | -1.43066 | 4.77E-03 | 4.30E-02 |
| 194 | XP_003014781.1 | TRINITY_DN518_c0_g1_i3 | -1.4328 | 1.66E-07 | 1.73E-05 |
| 195 | F2PIH6 | TRINITY_DN1260_c0_g1_i2 | -1.44197 | 4.49E-06 | 2.29E-04 |
| 196 | XP_003015296.1 | TRINITY_DN120_c0_g1_i3 | -1.44325 | 6.44E-13 | 2.62E-10 |
| 197 | NA | TRINITY_DN1964_c0_g3_i1 | -1.45398 | 2.46E-04 | 4.81E-03 |
| 198 | NA | TRINITY_DN34_c1_g2_i1 | -1.45446 | 1.35E-04 | 3.09E-03 |
| 199 | NA | TRINITY_DN3504_c0_g1_i1 | -1.45844 | 4.36E-03 | 4.00E-02 |
| 200 | NA | TRINITY_DN1296_c1_g1_i1 | -1.45877 | 2.61E-03 | 2.73E-02 |
| 201 | XP_003232439.1 | TRINITY_DN246_c11_g1_i3 | -1.4684 | 7.63E-13 | 2.88E-10 |
| 202 | F2Q181 | TRINITY_DN186_c2_g1_i2 | -1.5255 | 1.83E-13 | 8.35E-11 |
| 203 | F2PPU6 | TRINITY_DN91_c0_g1_i11 | -1.53553 | 2.79E-06 | 1.60E-04 |
| 204 | F2PX58 | TRINITY_DN3660_c1_g1_i1 | -1.53587 | 5.98E-03 | 5.00E-02 |
| 205 | F2Q3N2 | TRINITY_DN238_c1_g3_i2 | -1.54969 | 4.42E-10 | 1.17E-07 |
| 206 | NA | TRINITY_DN8791_c0_g2_i1 | -1.56161 | 2.40E-03 | 2.56E-02 |
| 207 | NA | TRINITY_DN11830_c0_g2_i2 | -1.56679 | 2.94E-03 | 2.97E-02 |
| 208 | NA | TRINITY_DN208_c1_g2_i1 | -1.58803 | 1.01E-05 | 4.14E-04 |
| 209 | F2PHP3 | TRINITY_DN2513_c0_g1_i1 | -1.59161 | 4.30E-03 | 3.99E-02 |
| 210 | F2PJA2 | TRINITY_DN730_c0_g1_i2 | -1.59672 | 2.24E-06 | 1.33E-04 |
| 211 | F2Q189 | TRINITY_DN277_c0_g2_i17 | -1.62348 | 2.18E-05 | 7.90E-04 |
| 212 | F2PP56 | TRINITY_DN125_c2_g1_i1 | -1.62692 | 4.22E-06 | 2.17E-04 |
| 213 | XP_002625200.1 | TRINITY_DN381_c11_g1_i3 | -1.63267 | 1.10E-05 | 4.48E-04 |
| 214 | XP_003013021.1 | TRINITY_DN763_c0_g3_i4 | -1.65113 | 4.86E-06 | 2.42E-04 |
| 215 | F2PY35 | TRINITY_DN1116_c0_g1_i8 | -1.65914 | 2.05E-05 | 7.47E-04 |
| 216 | F2PK65 | TRINITY_DN186_c7_g1_i16 | -1.67685 | 1.29E-09 | 2.72E-07 |
| 217 | F2PKI1 | TRINITY_DN221_c10_g1_i7 | -1.69523 | 9.44E-08 | 1.09E-05 |
| 218 | F2PIV6 | TRINITY_DN771_c0_g2_i2 | -1.69982 | 6.56E-04 | 1.03E-02 |
| 219 | XP_003237197.1 | TRINITY_DN1575_c0_g1_i5 | -1.69994 | 4.66E-04 | 7.93E-03 |
| 220 | F2Q0V9 | TRINITY_DN1859_c0_g1_i1 | -1.70551 | 5.00E-15 | 3.30E-12 |
| 221 | F2PR50 | TRINITY_DN1281_c0_g1_i1 | -1.72184 | 1.78E-03 | 2.05E-02 |
| 222 | NA | TRINITY_DN1163_c2_g1_i1 | -1.7255 | 3.49E-06 | 1.90E-04 |
| 223 | F2Q4W2 | TRINITY_DN261_c13_g1_i1 | -1.72593 | 4.30E-03 | 3.99E-02 |
| 224 | F2PGW6 | TRINITY_DN42_c1_g2_i2 | -1.72667 | 1.33E-06 | 8.29E-05 |
| 225 | NA | TRINITY_DN201_c4_g2_i1 | -1.74622 | 1.22E-07 | 1.31E-05 |
| 226 | XP_003011067.1 | TRINITY_DN2334_c0_g1_i1 | -1.76606 | 4.34E-05 | 1.28E-03 |
| 227 | XP_003012764.1 | TRINITY_DN406_c4_g1_i1 | -1.79163 | 1.36E-03 | 1.69E-02 |
| 228 | XP_003236947.1 | TRINITY_DN1255_c1_g1_i1 | -1.8418 | 1.34E-04 | 3.07E-03 |
| 229 | F2Q4R0 | TRINITY_DN186_c3_g1_i6 | -1.8531 | 1.97E-05 | 7.40E-04 |
| 230 | XP_003236173.1 | TRINITY_DN186_c13_g1_i1 | -1.93375 | 6.60E-06 | 2.98E-04 |
| 231 | NA | TRINITY_DN222_c0_g4_i2 | -1.94758 | 1.03E-08 | 1.65E-06 |
| 232 | XP_003238068.1 | TRINITY_DN1178_c0_g1_i1 | -1.97271 | 2.89E-04 | 5.50E-03 |
| 233 | XP_003013065.1 | TRINITY_DN545_c10_g1_i1 | -2.00366 | 2.46E-04 | 4.81E-03 |
| 234 | F2Q4S5 | TRINITY_DN756_c2_g1_i2 | -2.0135 | 5.13E-07 | 4.11E-05 |
| 235 | F2PV07 | TRINITY_DN3227_c0_g2_i1 | -2.04259 | 7.50E-06 | 3.33E-04 |
| 236 | XP_003013969.1 | TRINITY_DN1209_c0_g1_i11 | -2.05113 | 1.78E-14 | 1.05E-11 |
| 237 | F2PRL3 | TRINITY_DN1105_c2_g4_i4 | -2.10715 | 4.17E-06 | 2.16E-04 |
| 238 | F2PUA0 | TRINITY_DN2278_c0_g1_i2 | -2.12411 | 9.74E-05 | 2.42E-03 |
| 239 | NA | TRINITY_DN2618_c0_g1_i1 | -2.14028 | 1.05E-03 | 1.43E-02 |
| 240 | F2PJX8 | TRINITY_DN170_c0_g2_i3 | -2.15289 | 6.24E-09 | 1.09E-06 |
| 241 | F2PWL6 | TRINITY_DN208_c1_g1_i1 | -2.16837 | 4.03E-17 | 5.33E-14 |
| 242 | F2Q262 | TRINITY_DN263_c0_g3_i6 | -2.34279 | 9.64E-17 | 1.02E-13 |
| 243 | XP_003233947.1 | TRINITY_DN1614_c0_g1_i2 | -2.35174 | 7.89E-06 | 3.45E-04 |
| 244 | F2PY33 | TRINITY_DN982_c0_g1_i1 | -2.41108 | 1.45E-07 | 1.53E-05 |
| 245 | XP_003013882.1 | TRINITY_DN521_c0_g1_i11 | -2.41498 | 1.90E-07 | 1.89E-05 |
| 246 | XP_003013966.1 | TRINITY_DN1209_c0_g2_i9 | -2.58345 | 1.89E-13 | 8.35E-11 |
| 247 | F2Q3E4 | TRINITY_DN1590_c0_g1_i1 | -2.77396 | 2.73E-07 | 2.49E-05 |
| 248 | F2PY29 | TRINITY_DN521_c2_g1_i7 | -2.9045 | 1.14E-06 | 7.48E-05 |
| 249 | F2PRN1 | TRINITY_DN513_c1_g1_i8 | -3.20286 | 8.32E-07 | 6.12E-05 |
| 250 | XP_003236177.1 | TRINITY_DN1508_c0_g1_i1 | -3.24349 | 2.30E-07 | 2.17E-05 |
| 251 | F2Q4W8 | TRINITY_DN761_c0_g1_i1 | -3.35865 | 3.48E-17 | 5.33E-14 |
| 252 | NA | TRINITY_DN342_c0_g1_i2 | -3.36108 | 3.07E-06 | 1.71E-04 |
| 253 | XP_003238217.1 | TRINITY_DN342_c0_g2_i1 | -3.43831 | 3.74E-08 | 5.07E-06 |
| 254 | XP_003231885.1 | TRINITY_DN69_c0_g1_i1 | -3.66508 | 1.09E-06 | 7.31E-05 |
| 255 | F2PTZ5 | TRINITY_DN1820_c0_g2_i2 | -4.07818 | 8.97E-08 | 1.07E-05 |
| 256 | NA | TRINITY_DN2368_c0_g1_i2 | -4.19368 | 1.51E-08 | 2.35E-06 |
| 257 | F2PTU3 | TRINITY_DN8418_c0_g1_i1 | -5.18118 | 5.73E-06 | 2.66E-04 |
| 258 | XP_003237525.1 | TRINITY_DN1343_c0_g2_i1 | -5.19275 | 1.74E-17 | 4.61E-14 |
| 259 | XP_003012142.1 | TRINITY_DN1706_c0_g2_i1 | -5.80703 | 4.86E-15 | 3.30E-12 |
| 260 | EHJ70060 | TRINITY_DN1916_c0_g1_i1 | -7.52302 | 6.79E-05 | 1.80E-03 |

Table 10. Differential proteins.

| No. | UniProtKB | Protein Name | logFC | P-value |
| --- | --- | --- | --- | --- |
| 1 | C5HA31 | Ribosomal protein S5 | 4.169007 | 3.56E-49 |
| 2 | A0A6C0PQX8 | Translation elongation factor 1-alpha | 3.603976 | 7.98E-111 |
| 3 | A0A6H0C9I0 | Squalene monooxygenase | 2.862456 | 2.02E-30 |
| 4 | A0A3G2BRP4 | Cytochrome b | 2.65391 | 8.22E-42 |
| 5 | Q9HGX8 | Alpha-crystallin-related protein | 2.158522 | 1.55E-40 |
| 6 | C4MCE9 | Glyceraldehyde-3-phosphate dehydrogenase | 1.834137 | 1.90E-33 |
| 7 | H6X196 | Beta-tubulin | 1.604074 | 1.57E-08 |
| 8 | A0A0M4KE71 | Dipeptidyl-peptidase V | 1.588866 | 8.52E-26 |
| 9 | H3K2X5 | Tryptophan synthase | 1.495574 | 4.68E-21 |
| 10 | A0A2I6PMV5 | Ribosomal protein | 1.482792 | 4.28E-23 |
| 11 | C5HA37 | NADH dehydrogenase subunit 2 | 1.363462 | 6.31E-12 |
| 12 | A0A7T7WX11 | Metallocarboxypeptidase B | 1.117096 | 3.15E-13 |
| 13 | C5HA40 | NADH-ubiquinone oxidoreductase chain 1 | 1.038589 | 1.57E-06 |
| 14 | A0A0K2QQ36 | Succinate dehydrogenase | -1.41029 | 1.51E-20 |
| 15 | A0A4D6CK33 | Heat shock protein 70 | -1.49637 | 2.70E-07 |
| 16 | C9K6C4 | Actin | -1.63154 | 1.94E-24 |
| 17 | X5CH36 | 2-phosphoglycerate dehydratase | -2.2875 | 3.26E-51 |
